# The Arabidopsis 2′-O-Ribose-Methylation and Pseudouridylation Landscape of rRNA in Comparison to Human and Yeast

**DOI:** 10.3389/fpls.2021.684626

**Published:** 2021-07-26

**Authors:** Deniz Streit, Enrico Schleiff

**Affiliations:** ^1^Department of Biosciences, Molecular Cell Biology of Plants, Goethe University, Frankfurt, Germany; ^2^Frankfurt Institute for Advanced Studies (FIAS), Frankfurt, Germany

**Keywords:** snoRNA, *A. thaliana*, rRNA, ribosome, 2cpsdummy′-O-ribose-methylation, pseudouridylation

## Abstract

Eukaryotic ribosome assembly starts in the nucleolus, where the ribosomal DNA (rDNA) is transcribed into the 35S pre-ribosomal RNA (pre-rRNA). More than two-hundred ribosome biogenesis factors (RBFs) and more than two-hundred small nucleolar RNAs (snoRNA) catalyze the processing, folding and modification of the rRNA in *Arabidopsis thaliana*. The initial pre-ribosomal 90S complex is formed already during transcription by association of ribosomal proteins (RPs) and RBFs. In addition, small nucleolar ribonucleoprotein particles (snoRNPs) composed of snoRNAs and RBFs catalyze the two major rRNA modification types, 2′-O-ribose-methylation and pseudouridylation. Besides these two modifications, rRNAs can also undergo base methylations and acetylation. However, the latter two modifications have not yet been systematically explored in plants. The snoRNAs of these snoRNPs serve as targeting factors to direct modifications to specific rRNA regions by antisense elements. Today, hundreds of different sites of modifications in the rRNA have been described for eukaryotic ribosomes in general. While our understanding of the general process of ribosome biogenesis has advanced rapidly, the diversities appearing during plant ribosome biogenesis is beginning to emerge. Today, more than two-hundred RBFs were identified by bioinformatics or biochemical approaches, including several plant specific factors. Similarly, more than two hundred snoRNA were predicted based on RNA sequencing experiments. Here, we discuss the predicted and verified rRNA modification sites and the corresponding identified snoRNAs on the example of the model plant *Arabidopsis thaliana*. Our summary uncovers the plant modification sites in comparison to the human and yeast modification sites.

## Introduction

Ribosome biogenesis is an essential biochemical process in all existing organisms. The formation of functional ribosomes involves a huge number of different RNAs and proteins, which have to act in a defined order. These factors catalyze various steps during the maturation of ribosomal RNA (rRNA) from the initial precursor including, their folding, modifications of the rRNA and the assembly of ribosomal proteins. For model systems like yeast, the understanding of molecular events during ribosome biogenesis are already well described. For example, comprehensive number of ribosome assembly factors and their functions, in addition to availability of high resolution ribosome structure, paved the way for in-depth analysis of ribosome maturation in yeast (Woolford and Baserga, 2013; Klinge and Woolford, 2019). While for the same processes in plant systems, many aspects are yet to be given a detailed account. For the analysis of the processes in plants, *Arabidopsis thaliana* has become the model plant for the examination of ribosome biogenesis next to crop plants like wheat and rice (Armache et al., 2010b; Hang et al., 2018).

**FIGURE 1:**
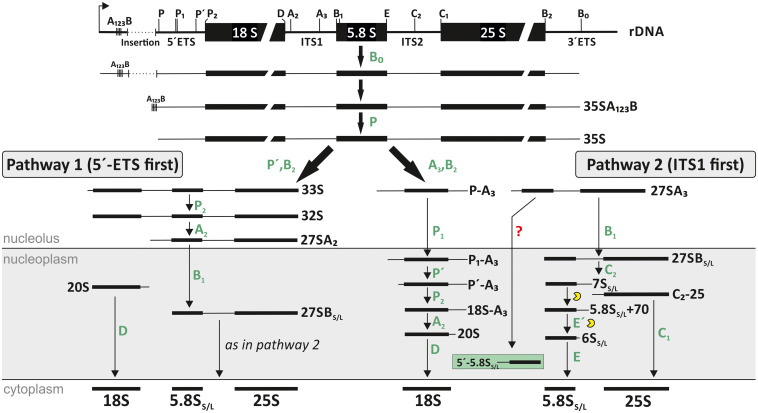
Ribosomal RNA (rRNA) processing pathway in *A. thaliana*. After transcription the 35S pre-rRNA is formed. After cleavage at site P, the further processing depends on the next cleavage site. While for pathway 1 the next cleavage occurs in the 5′-ETS (similar to the main pathway in yeast), the second and majorly used pathway is characterized by a first cleavage in the ITS1 region (similar to the human pathway). The location of each existing precursor of Arabidopsis is shown. However, the exact localization of 27S and 18S precursors are not fully analyzed yet. The figure was modified from Weis et al. (2015a).

**FIGURE 2:**
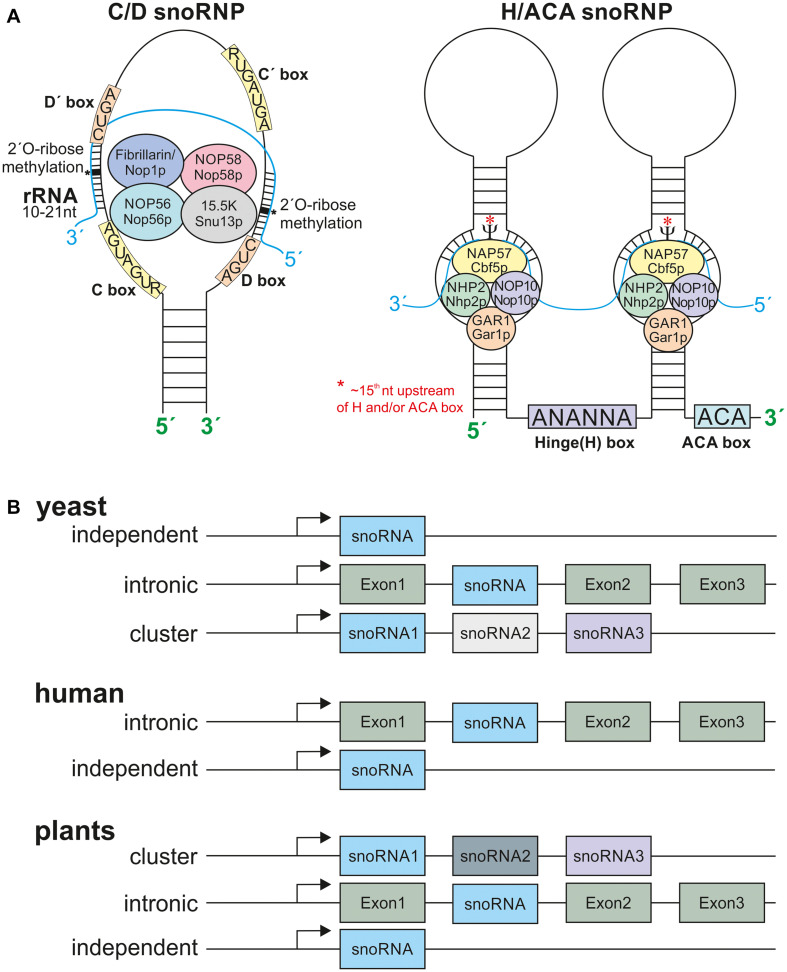
The snoRNPs and snoRNA localization. **(A)** Depicted are the C/D box snoRNPs and H/ACA snoRNPs. The C/D box snoRNPs contain the conserved C (RUGAUGA) and D (CUGA) boxes and the two less conserved C’ and D’-boxes. The C/D box snoRNPs are composed of the methyltransferase fibrillarin/Nop1p, NOP56/Nop56p, Nop58/Nop58p, and 15.5K/Snu13p. The rRNA has a 10–21 nt long complementary site to the according snoRNA. The 2′-O-ribose-methylation takes place 5 nt downstream of the D or D’ box (asterisks). The H/ACA box snoRNPs are composed of at least one stem loop. The Hinge Box (ANANNA) is located between two stem loops and the ACA box at the 3′ end of the snoRNA. The pseudouridine synthase NAP57/Cbf5p is modifying approx. the 15th nucleotide (asterisks) of the rRNA upstream of the H and or ACA box, further the proteins NHP2/Nhp2p, Nop10/Nop10p, and GAR1/Gar1p are required. **(B)** The snoRNA gene organization in different eukaryotes. Yeast snoRNAs are mainly localized in independent regions and lesser in intronic and polycistronic (cluster) regions. The human snoRNA gene organization is mostly intronic with few independent genes and plant snoRNAs are mostly located within clusters of many snoRNAs. Only very few examples for intronic and independent gene organization are known. For references see main text.

The maturation of 80S ribosomes is coordinated between three different compartments of the cell. It begins with the transcription of the 35S pre-rRNA by RNA-polymerase I in the nucleolus (Tsang et al., 2003; Henras et al., 2008; Missbach et al., 2013; Hellmann, 2020). The 35S pre-rRNA consists of the three rRNAs 18S, 5.8S, and 25S. The 18S and 5.8S rRNA are separated by the internal transcribed spacer 1 (ITS1), 5.8S and 25S rRNA by the internal transcribed spacer 2 (ITS2) and the three maturing rRNAs are additionally flanked by the 5′- and 3′-external transcribed spacers (ETSs); (Tollervey and Kiss, 1997; Lafontaine, 2015; Weis et al., 2015b; Figure 1). This precursor is subsequently processed and modified. The maturation of the rRNA is assisted by ribosome biogenesis factors (RBFs) and small nucleolar RNAs (snoRNAs) (Figure 2A). Initially, the 90S particle formation is followed by subsequent splitting into pre-40S and pre-60S particles. The maturation of these particles occurs in the nucleolus, nucleoplasm and in the cytosol (Figure 1). During the maturation of ribosomal subunits, the precursors of 18S, 5.8S, and 25S rRNA are processed, folded and modified, and the final steps occur in the cytoplasm before final assembly of the 80S ribosomes (Henras et al., 2008; Palm et al., 2019; Sáez-Vásquez and Delseny, 2019). In addition to the rRNAs transcribed on the 35S transcript, the large ribosomal subunit (LSU) contains a 5S rRNA. This rRNA is transcribed independently in the nucleus by RNA-polymerase III (Henras et al., 2008; Missbach et al., 2013; Bassham and MacIntosh, 2017). The 5S rRNA forms the 5S RNP together with the ribosomal proteins L5 and L18, which associates with the 60S pre-ribosomal particle in the nucleoplasm (Leidig et al., 2014; Lafontaine, 2015).

To date, more than 200 RBFs and more than 200 snoRNAs were described to regulate ribosome maturation. In plants, the inventory for both has been established by a combination of experimental evidence and bioinformatics prediction (Barneche et al., 2001; Brown et al., 2001; Qu et al., 2001; Chen C. L. et al., 2003; Chen and Wu, 2009; Kim et al., 2010; Liu et al., 2013; Palm et al., 2016; Azevedo-Favory et al., 2020). Considering the importance of rRNA modifications for proper processing and maturation of rRNA but also for the function of ribosomes, we discuss in the following the current knowledge on the rRNA modifications and snoRNAs in Arabidopsis and compare these with the human and yeast modifications sites.

## The Eukaryotic snoRNAs

Alike messenger RNA (mRNA) and transfer RNA (tRNA), rRNA is highly post-transcriptional modified (Sloan et al., 2017). For yeast, it could be shown that the loss of individual modifications within the rRNA is non-essential, while the lack of more than one modification site, especially in important regions of the ribosome has led to alterations in ribosomal processing but also rRNA processing can be affected (Liang et al., 2009; Demirci et al., 2010; Polikanov et al., 2015). Furthermore, different distributions of modification sites can be related to different cell type as reported for human ribosomes, where cancer cells carry a different subset of modifications (Natchiar et al., 2017). Nonetheless, in contrast it could be demonstrated that the loss of a single modification site in zebrafish can have harmful effects during the early development (Higa-Nakamine et al., 2012). However, only a small number of rRNA modification types are known. The two major modifications are 2′-O-ribose-methylation (2′-O-ribose-me), where a methyl group is attached to the 2′ hydroxyl-group of the ribose within nucleosides, and pseudouridylation involving the conversion of uridine to pseudouridine (Zhao and Yu, 2004; Ito et al., 2014). Recent studies in yeast showed, that the acetyltransferase Kre33 acetylates the sites ac^4^C1773 and ac^4^C1280 of the 18S rRNAs, which are guided by the two orphan C/D box snoRNAs snR4 and snR45 (Ito et al., 2014; Sharma et al., 2017b). Some snoRNAs like the abundant C/D box snoRNAs U3 and U14 are rather involved in pre-rRNA cleavage at the 5′-ETS site and are therefore involved in 18S rRNA production (Brown and Shaw, 1998; Venema and Tollervey, 1999; Brown et al., 2003).

The 2′-O-ribose-me is the most frequently occurring modification within RNA and can be important for RNA degradation. For example, it was observed that miRNA and siRNAs lacking 2′-O-ribose-me on the 3′ terminal ribose are exposed to degradation (Zhao et al., 2012). In addition, 2′-O-ribose-me defines local secondary structures (Filippova et al., 2017). Likewise, the isomerization of uridine to pseudouridine confers stability of hairpins by base stacking (Desaulniers et al., 2008; Filippova et al., 2017). Modifications often occur in functionally relevant areas of the ribosomes such as A, P, and E sites, the peptidyl transferase center (PTC) and the intersubunit bridge (Decatur and Fournier, 2002; Watkins and Bohnsack, 2012). Remarkably, RNA and especially rRNA modifications appear to be altered during development in addition to environmental changes, which could indicate ribosome heterogeneity (Sloan et al., 2017).

Small nucleolar RNAs are small RNA molecules essential for the regulation and guidance of the post-transcriptional modifications of rRNA, tRNA, and snRNAs (Kiss, 2001; Kruszka et al., 2003; Chow et al., 2007). SnoRNAs exist in eukaryotes and archaea, but not in bacteria (Terns and Terns, 2002; Bhattacharya et al., 2016). Because of their importance for rRNA folding and modification, they are often localized in the nucleus where processing and modification of rRNA takes place (Kiss-László et al., 1998). They are re-localized to the cytoplasm in response to stress, which has so far only been described in yeast (Holley et al., 2015). Whether this holds true for eukaryotes in general needs to be elucidated.

The sizes of snoRNAs vary between 60 and 300 nucleotides (nt; Falaleeva et al., 2017) and they are mostly transcribed by RNA-polymerase II (Qu et al., 2015). Nevertheless, in cases of the U3 gene in plants and in dicistronic tRNA-snoRNA genes, RNA-Polymerase III is responsible for the transcription (Dieci et al., 2009). The snoRNA gene organization varies between organisms. Most snoRNAs in yeast are independently encoded, and only a minority is localized in intronic regions or in cluster with other snoRNA genes (Brown et al., 2003; Figure 2B). The majority of snoRNAs in humans are organized in intronic regions, and only few are encoded as independent genes (Figure 2B). In plant genomes snoRNAs are encoded either independently, in intronic regions or in intronic gene clusters as shown in rice (Figure 2B; Leader et al., 1997; Brown et al., 2003). In addition, snoRNAs are also organized in dicistronic tRNA-snoRNAs or snoRNA-miRNA clusters as described in *A. thaliana* and rice (Kruszka et al., 2003; Qu et al., 2015).

The snoRNAs are classified by the existence of conserved sequence motifs (Bachellerie and Cavaillé, 1997; Weinstein and Steitz, 1999). The so called C/D box and H/ACA box snoRNAs form the two major classes, while some minor classes have been identified as well. The C/D box snoRNAs are characterized by a “C box” with a consensus sequence RUGAUGA (R stands for any purine) and a “D box” (consensus sequence: CUGA) (Figure 2A). Frequently, these snoRNAs contain additional, less conserved boxes annotated as C’ and D’. The conserved C and D boxes fulfill a multitude of functions and are amongst necessary for the snoRNA import into the nucleolus (Samarsky et al., 1998; Newman et al., 2000; Bertrand and Fournier, 2013). In contrast, binding to the rRNA target region is accomplished by one or two antisense elements of about 10–21 nt positioned upstream of the D or D’-boxes. In most cases, the complement to the fifth nucleotide of this element is modified in the rRNA (Barneche et al., 2001; Kiss, 2001; Kruszka et al., 2003). The secondary structure of C/D box snoRNA is characterized by a K-turn motif that brings the C and D box (C’ and D’) in proximity through stem loop formation, and by guide elements carrying the antisense sequences (Matera et al., 2007). The C/D box snoRNAs are components of small nucleolar ribonucleoprotein particles (snoRNPs) that in addition consists of described four core proteins fibrillarin (methyltransferase; Nop1p in yeast), NOP58 (Nop58p in yeast), NOP56 (Nop56p in yeast), and 15.5K (Snu13p in yeast) (Rodor et al., 2011; Huang et al., 2016; Figure 2A).

The hinge box (H-box: sequence ANANNA, N stands for any nucleotide) and the 3′ terminal located ACA box characterize the H/ACA box snoRNA family (Brown and Shaw, 1998; Kiss, 2001). The H and ACA boxes are required for nucleolar import, for example (Bertrand and Fournier, 2013). H/ACA snoRNPs form a hairpin-hinge-hairpin-tail structure with the tail and the hinge region being single stranded (Dragon et al., 2006). The nucleotide to be modified is positioned about 15 nt upstream of the ACA or hinge motif, respectively (Lindsay et al., 2013; Figure 2A). Alike the C/D box snoRNAs, H/ACA box snoRNAs are components of snoRNPs. However, the known snoRNPs containing an H/ACA box snoRNA consist of the proteins dyskerin/NAP57 (pseudouridine synthase; Cbf5p in yeast), NHP2 (Nhp2p), NOP10 (Nop10p), and GAR1 (Gar1p) (Rodor et al., 2011; Figure 2A).

Another minor class of snoRNAs unifies the mitochondrial RNA processing (MRP)-RNAs, a snoRNA family which lacks conserved boxes, but harbors rRNA processing activity (Bertrand and Fournier, 2013). Additional snoRNAs without typical motifs are deposited in plant snoRNA databases as well (Yoshihama et al., 2013).

## The snoRNAs in Plants

Since the first discoveries of snoRNAs in 1970’s (Reddy et al., 1974, 1979) different studies targeted the identification of plant snoRNAs by experimental approaches. Early on, the snoRNAs U3, U14, and U49 were identified in plants based on similarity to the snoRNAs of yeast and vertebrates (Kiss et al., 1991; Leader et al., 1994, 1997). Remarkably, U3 in plants is transcribed by the RNA polymerase III and possess a different capping than found for U3 in yeast or human (Kiss et al., 1991). By dot-matrix analysis of Fib1 and Fib2, the plant-specific snoRNAs U60.1f and U60.2f were discovered (Barneche et al., 2000).

After the release of the *A. thaliana* genome (Kaul et al., 2000) snoRNAs were identified by computational strategies searching for C/D box characteristics, rRNA complementarities or other structural attributes (Zhou et al., 2000; Barneche et al., 2001; Brown et al., 2001; Qu et al., 2001). The next boost for the discovery of plant snoRNAs came by RNomics on either total RNA from different tissues or from the nucleolar RNA of *A. thaliana* (Marker et al., 2002; Kim et al., 2010; Streit et al., 2020) and by re-analysis of existing small RNA datasets of different *A. thaliana* tissues and growth stages (Chen and Wu, 2009). This analysis was initially focused on Arabidopsis and was then extended to *Oryza sativa* (Chen C. L. et al., 2003; Liu et al., 2013). Today, 10,654 different H/ACA box snoRNA genes and 6064 different C/D box snoRNA genes are deposited in the database snOPY (Yoshihama et al., 2013).

In contrast to globally discovered snoRNAs, only a single plant snoRNA is functionally characterized. The C/D box type snoRNA HIDDEN TREASURE 2 (HID2) associates with 45S pre-rRNA but is not relevant for 2′-ribose methylation at position G2620 as this modification was not altered in an according mutant (Zhu et al., 2016). It was speculated that other snoRNAs might complement for HID2 function (Zhu et al., 2016), which needs to be verified. Thus, the analysis of the snoRNA function and the complementarity of the different snoRNA genes of the different families will be a major target of future research.

## The rRNA Modification in Plants

There are two major types of rRNA modifications, namely 2′-O-ribose-methylation and pseudouridylation. However, for yeast and human rRNAs, base methylations were additionally described (e.g., for yeast dimethylase Dim1p) (Lafontaine et al., 1995). Furthermore, yeast rRNA was also found to be acetylated (Ito et al., 2014; Sharma et al., 2017b). However, the latter two modification types have not been described so far in plants (Piekna-Przybylska et al., 2007).

Initially, the analysis of the individual snoRNAs was accompanied by the analysis of the rRNA modification sites, e.g., by primer extension analysis (Barneche et al., 2000). Recently, genome-wide pseudouridine sequencing verified predicted pseudouridine modifications in cytosolic and plastidic ribosomes (Sun et al., 2019). At the same time, this approach led to the discovery of yet unknown modification sites as well (Sun et al., 2019). Remarkably, the ITS1 separating the 18S rRNA from 5.8S is modified as well (Sun et al., 2019). However, future studies are required to explore whether this is a unique modification or whether ITS1, ITS2 and the ETS regions are generally modified, and to understand the role of modifications of the pre-rRNA. A complementary analysis using RiboMethSeq for detection of 2′-O-ribose-me modifications yielded novel modification sites as well (Azevedo-Favory et al., 2020).

Different approaches exploring the modifications of the rRNA in Arabidopsis yielded a total of 321 rRNA modification sites (Barneche et al., 2001; Qu et al., 2001; Chen and Wu, 2009; Kim et al., 2010; Sun et al., 2019; Azevedo-Favory et al., 2020; Streit et al., 2020). A total of 79 2′-O-ribose-me and 43 pseudouridylation sites were assigned for the 18S rRNA, of which 44 2′-O-ribose-me and 28 pseudouridylation sites were experimentally confirmed. For 25S rRNA, 132 2′-O-ribose-me and 64 pseudouridylation sites are proposed, of which 86 and 51, respectively, are experimentally confirmed. For 5.8S rRNA, three sites carrying 2′-O-ribose-me were predicted due to antisense elements found in three snoRNAs of which two are experimentally confirmed. Accordingly, a recent study confirmed the predicted U22 pseudouridylation and mapped a new site at U78 (Sun et al., 2019).

It has to be considered that the existing discrepancy between detected and predicted modification sites might result from a variability of the modification pattern in ribosomes of one cell, in different tissues, at different developmental stages or in response to environmental changes as discovered for other species named ribosome heterogeneity (Sloan et al., 2017). However, as the ribosome turn-over is comparatively slow, alterations in rRNA modifications are considered to be more meaningful for long-term changes (Ferretti and Karbstein, 2019). Although the final annotation and confirmation of predicted sites requires further research, in here the predicted sites are discussed as well. In the following sections, the positioning of the modifications in the rRNA, for selected modifications the function and the required snoRNAs are discussed.

## A View on the rRNA Modification Sites and snoRNAs in *Arabidopsis thaliana*

### A View on the Modifications in Plant 5.8S rRNA

**FIGURE 3:**
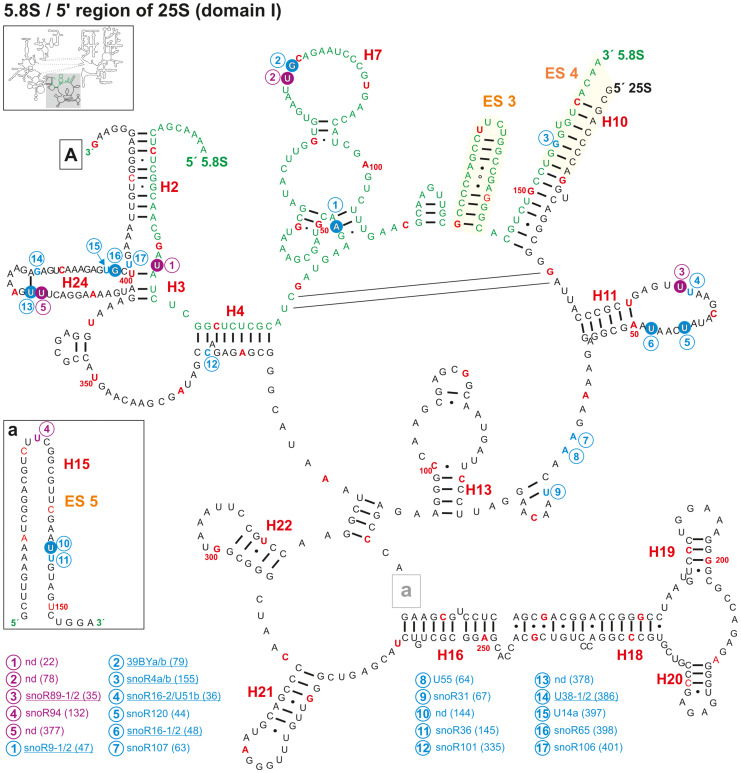
Secondary structure diagram of the 5.8S rRNA and domain I of 25S rRNA of *A. thaliana*. There is no experimental determined secondary structure map of the rRNA of *A. thaliana*. Hence, the secondary map of 5.8S (letters in green) and 25S (letters in black) was created based on the RNAcentral database (The RNAcentral Consortium, 2019) and http://www.rna.icmb.utexas.edu. The positions for predicted (blue letter) and verified 2′-O-ribose-me (white letter in blue circle) sites and the predicted (violet letter) and verified positions for pseudouridylation (white letter in violet circle) are shown. If several snoRNAs are annotated to target the same site, the name is underlined. Analyses were conducted by using the snoRNA databases snOPY (Yoshihama et al., 2013) and the plant snoRNA DB (Brown et al., 2003). Predicted and verified positions for 2′-O-ribose-methylations and pseudouridylations were obtained from Barneche et al. (2001), Brown et al. (2001), Qu et al. (2001), Sun et al. (2019), Azevedo-Favory et al. (2020). Every tenth nucleotide is marked in red and every 50th nucleotide is labeled with the according number. Framed small letters indicate the position of the structures shown separately and framed large letters indicate the position of the connections in subsequent images (for A see Figure 4). The sequence used for the secondary structure map refers to the sequence of 5.8S and 25S of chromosome 2. The number in brackets correspond to the modified nucleotide position. A small illustration of the whole 60S rRNA secondary structure highlighting the according region is enclosed.

For the 5.8S rRNA, three 2′-O-ribose-me sites were predicted (Figure 3 and Supplementary Table 1), of which two sites were mapped by primer extension (Barneche et al., 2001; Brown et al., 2001; Qu et al., 2001). Additionally, two pseudouridylation sites in 5.8S could be mapped as well (Sun et al., 2019). Thus, the modification of the 5.8S rRNA in *A. thaliana* is more similar to human with four modifications (two 2′-O-ribose-me and two pseudouridylation sites) than to yeast, where only a single Ψ-site at U73 is known so far (Piekna-Przybylska et al., 2007). However, the Ψ-site in yeast exists in *A. thaliana* at position Ψ78, although this Ψ-site was found at the adjacent uracil in Arabidopsis (Figure 3). The H/ACA box snoRNA snR43 targeting this site in yeast (Piekna-Przybylska et al., 2007) could not be identified in *A. thaliana*.

The sites Am47, Gm79, and Ψ78 are localized in the 5.8S secondary structure, which is formed by three bulges between the helices 5, 6, and 7 (Figure 3 and Supplementary Table 1). Worth mentioning, the 2′-O-ribose-me at Gm79 in *A. thaliana* (Figure 3) represents the Gm75 modification site in humans (Piekna-Przybylska et al., 2007). Remarkably, in human ribosomes the region which includes helices 5, 6, and 7 is sandwiched between uL26, L35/uL29, L37, and eL39 (Khatter et al., 2015) and the structure changes between the mRNA free and the mRNA bound state (Graifer et al., 2005). A similar structure was obtained in plant ribosomes, where the helices 5, 6, and 7 are sandwiched by L24, L29, L37e, and L39e (Armache et al., 2010a). Moreover, L29 was identified as one of the ribosomal proteins with diurnal alteration of the phosphorylation state in Arabidopsis (Turkina et al., 2011). This suggest that the modification in this region of the 5.8S rRNA might be important for the ribosomal activity in translation (Gulay et al., 2017) or for the ribosomal translation elongation (translocation), which was found in cell-free extracts to be under the regulation of the 5.8S rRNA as well (Elela and Nazar, 1997).

In addition, one modification is found in the bulge between helix 2 and helix 3, and one in helix 10. All three helices are formed by base pairing between 5.8S and 25S rRNA (Figure 3) and are deeply buried in the ribosomal structure in the human ribosomes (Khatter et al., 2015). Thus, it is likely that the modifications are required for stabilizing the structure of the ribosomes. Interestingly, the predicted 2′-ribose-O-me site at position Gm155, which hypothetically is targeted by snoR4a/4b, could not be confirmed by radiographic labeling of modified nucleotides in wheat-embryo (Lau et al., 1974), suggesting that this snoRNA is probably not involved in the modification but rather in rRNA processing in the ITS2 region (Brown et al., 2001). However, it is known that certain modifications of the eukaryotic 5.8S are tissue specific (Nazar et al., 1975). Hence, it remains possible that modifications like Gm155 are only present in selected tissues or in developmental manner.

Furthermore, modifications such as 2′-O-ribose-methylations at Um14 in rat liver appeared to be present in a higher degree in the cytoplasmic fraction than in nuclear fractions (Nazar et al., 1980). However, the cellular distribution of the rRNA modifications in plant cells was not experimentally approached so far.

### A View on the Modifications in Arabidopsis 25S rRNA

**FIGURE 4:**
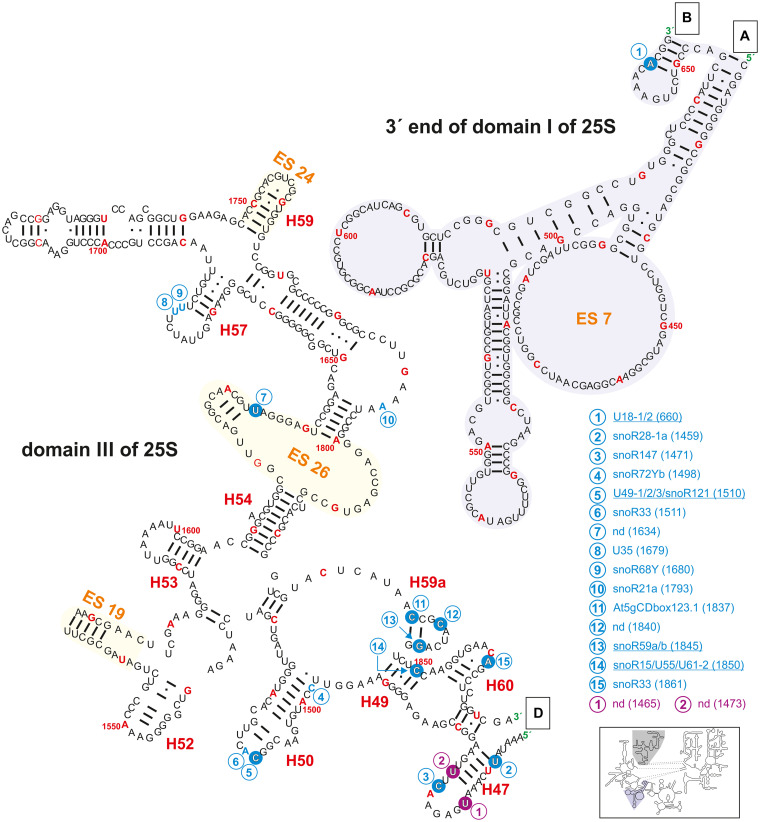
Secondary structure diagram of the 3′ end of domain I (gray background) as well as of domain III of 25S rRNA of *A. thaliana*. The image is shown according to the legend for Figure 3. Framed large letters indicate the position of the connections in subsequent images (for A see Figure 3; for B see Figure 6; for D see Figure 5). The number in brackets correspond to the modified nucleotide position. A small illustration of the whole 60S rRNA secondary structure highlighting the according region is enclosed.

**FIGURE 5:**
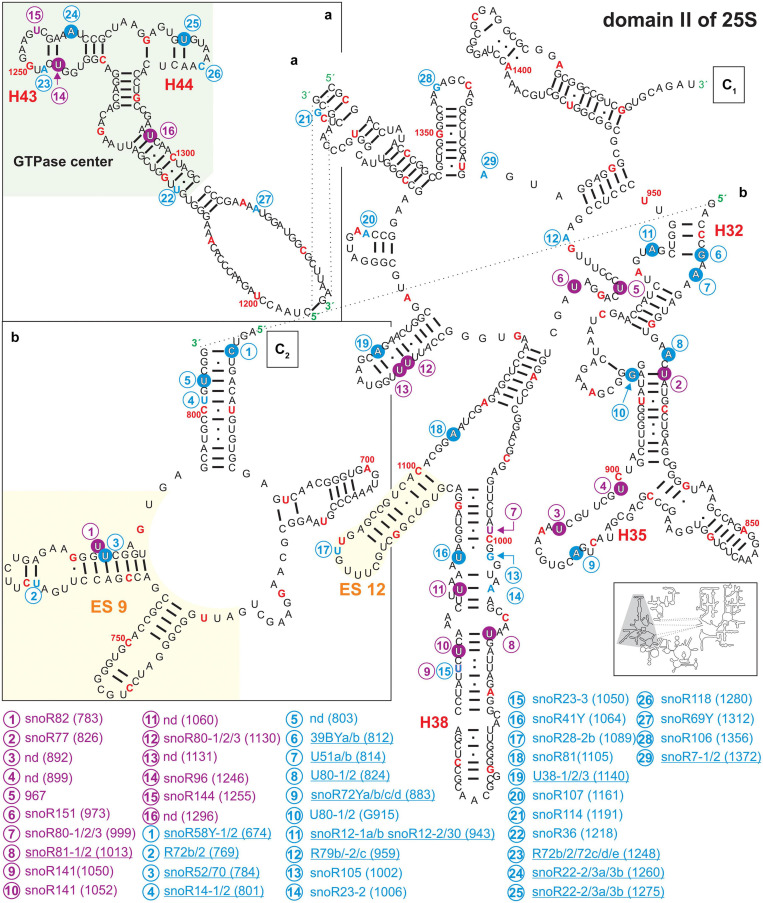
Secondary structure diagram of domain III of 25S rRNA of *A. thaliana*. The image is shown according to the legend for Figure 3. Framed large letters indicate the position of the connections in subsequent images (for C see Figure 6). PE annotates a pivoting element previously identified (Paci and Fox, 2015). The number in brackets correspond to the modified nucleotide position. A small illustration of the whole 60S rRNA secondary structure highlighting the according region is enclosed.

**FIGURE 6:**
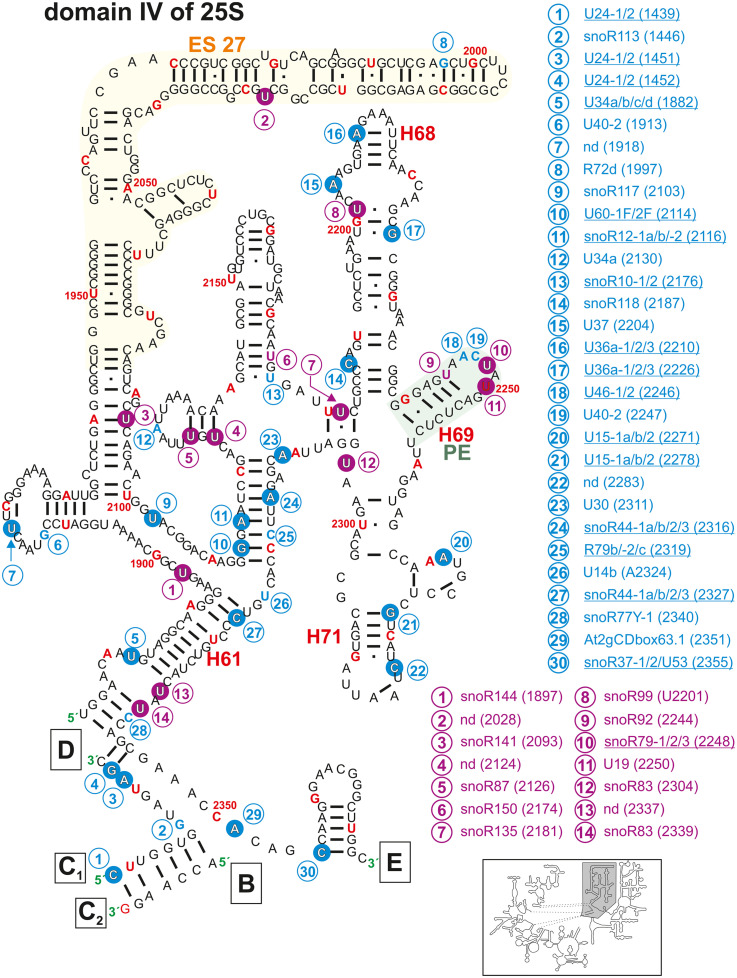
Secondary structure diagram of domain IV of 25S rRNA of *A. thaliana*. The image is shown according to the legend for Figure 3. Framed large letters indicate the position of the connections in subsequent images (for B see Figure 4; for C see Figure 5; for D see Figure 4; for E see Figure 7). The number in brackets correspond to the modified nucleotide position. A small illustration of the whole 60S rRNA secondary structure highlighting the according region is enclosed.

**FIGURE 7:**
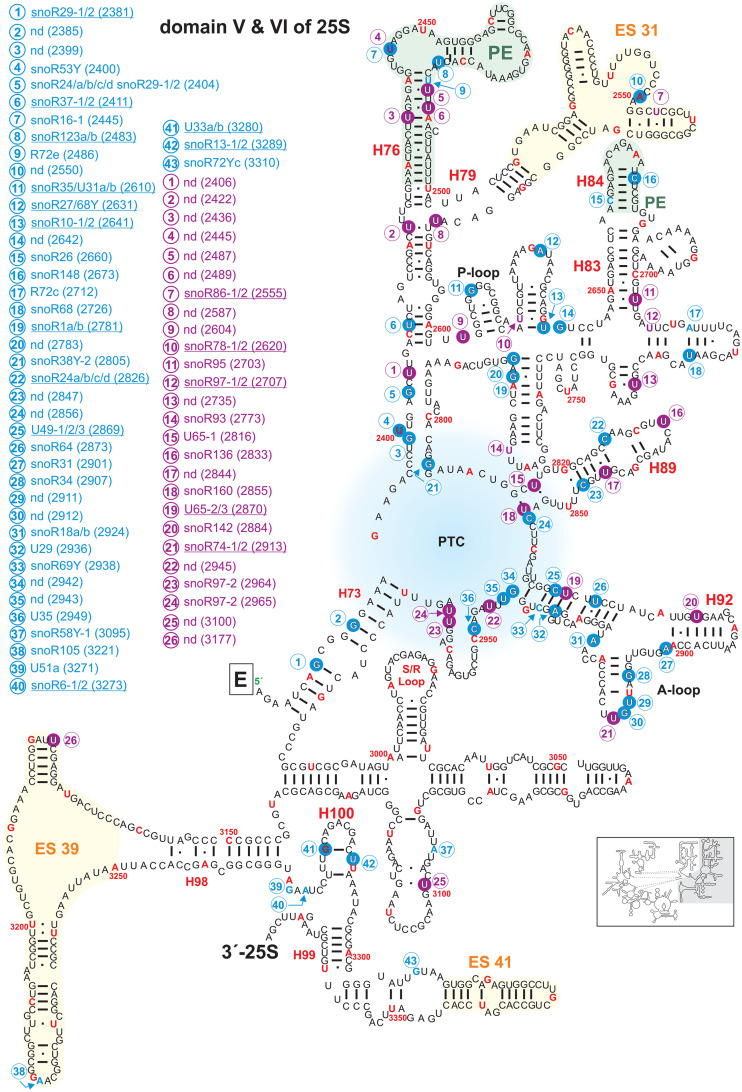
Secondary structure diagram of domain V and VI of 25S rRNA of *A. thaliana*. The image is shown according to the legend for Figure 3. Framed large letters indicate the position of the connections in subsequent images (for E see Figure 6). The region of the PTC is indicated in blue. The number in brackets correspond to the modified nucleotide position. A small illustration of the whole 60S rRNA secondary structure highlighting the according region is enclosed.

The 25S rRNA is the largest RNA within ribosomes and thus it contains numerous modifications. For better discussion, the 25S rRNA is dissected in here into five domains along the rRNA sequence. The 5′ region (bp 1–660) is assigned as domain I (Figures 3, 4), bp 660–1440 as domain II (Figure 5), bp 1440–1870 as domain III (Figure 4), bp 1870–2370 as domain IV (Figure 6) and the 3′ region (bp 2370–3375) is assigned as domain V/VI (Figure 7; Paci and Fox, 2015). The helical domains (H), the expansion segments (ES), and pivoting regions (PR) are in part numbered according to previous annotations (Taylor et al., 2009; Paci and Fox, 2015). Expansion segments are additional rRNA parts in eukaryotic rRNA compared to the prokaryotic rRNA. Though expansion segments can vary in their sequence and length but are rather conserved in their overall secondary structure (Ramesh and Woolford, 2016).

Domain I of the 25S rRNA contains five mapped and additionally nine predicted 2′-O-ribose-me sites (Figures 3, 4 and Supplementary Table 2), as well as two mapped and one predicted pseudouridylation site(s). The methylations can be found in helix 11, 15, and 24. Among others, helices 7, 18, 19, 20, and 24 surround the exit tunnel for the nascent polypeptide chain (Spahn et al., 2001). However, only in helix 24 two 2′-O-ribose-me sites and one pseudouridylation site were mapped (Figure 3). Accordingly, in human 28S rRNA, helix 24 carries a modification at site Am389 and Am391 (Sharma et al., 2017a), while no modification was found in domain I in yeast (Yang et al., 2016). In fact, signal recognition particles (SRPs) (Halic et al., 2004), which recognize specific sequences of nascent polypeptide chains from the translating ribosomes interact with the tip of helix 24 of the 25/28S rRNA (Beckmann et al., 2001). In turn one could conclude that human and plant ribosomes have evolved a similar mode of binding to such molecular mechanisms. More intriguingly, the presence or absence of modified nucleotides in this region could be used as a complex regulator for the interaction of such particles with the ribosomes of humans and plants. Furthermore, snoRNAs for helix 24 modification were identified for humans, but not for Arabidopsis (Supplementary Table 2). Hence, a stand-alone enzyme might be responsible for these modifications that could act in the cytoplasm of Arabidopsis.

Helix 11 of the 25S rRNA in Arabidopsis contains three positions with modifications (two 2′-O-ribose-me and one pseudouridylation site; Figure 3). However, this helix does not carry modifications in yeast or human (Piekna-Przybylska et al., 2007). Thus, it is highly likely that this helix has in plants or at least in Arabidopsis a special function within the 60S ribosomal subunits or even in the 80S ribosomes, which requires such modification.

The region annotated as expansion segment 7 (ES7) contains only one described modification site at Am660 which is targeted by the snoRNAs U18-1 and 2 (Figure 4 and Supplementary Table 2). The ES7 is known be localized at the ribosome surface and belongs to the largest expansion segments with the highest variability in eukaryotes (Ramos et al., 2016). It was found that proteins binding to ES7 were relevant for regulations upon environmental changes, 60S subunit biogenesis and transcription elongation (Ramos et al., 2016). In contrast, yeast and human ES7 of the 25/28S is substantially greater than in plants (Parker et al., 2018). Together with the fact that Arabidopsis ES7 carries a 2′-O-ribose-me it can be concluded that plants evolved a special way of regulating those important features during stress conditions as well as in other regulatory functions.

Domain II of Arabidopsis 25S rRNA carries 29 putative 2′-O-ribose-me sites of which 14 could be successfully mapped. For pseudouridylation, 16 sites were predicted of which 13 were mapped (Figure 5 and Supplementary Table 2). Domain II contains the GTPase center mainly composed of helix 43 and helix 44 (Figure 5), which is highly conserved in all ribosomes (Ryan and Draper, 1991). In *Escherichia coli*, this region including the ribosomal proteins L10 and L11 is involved in the regulation of GTP hydrolysis by the elongation factor G and TU (Egebjerg et al., 1990; Briones et al., 1998). The rRNA of the GTPase center in *A. thaliana* contains three mapped and two predicted modification sites, for which associated snoRNAs are assigned (Figure 5 and Supplementary Table 2). Interestingly the modifications seem to be unique for *A. thaliana* since this segment is not modified in human or yeast (Piekna-Przybylska et al., 2007).

In general, many of the mapped modification sites in domain II are localized in stem structures (Figure 3). The helices 27, 31, 32, 35, and 38 carry many modifications. In yeast and human, helix 38 is exceedingly pseudouridylated but does not contain any 2′-O-ribose-me modification (Piekna-Przybylska et al., 2007). In Arabidopsis, pseudouridylations and 2′-O-ribose-me modifications were predicted in this particular helix based on the detection of according snoRNAs. Moreover, three pseudouridylation sites and one 2′-O-ribose-me were experimentally confirmed in helix 38 of the rRNA of Arabidopsis. Nevertheless, for the pseudouridylation site at ψ1060 an according snoRNA could not be identified so far. Intriguingly, helix 38 is involved in the formation of the intersubunit bridge between the 60S and 40S subunit by interacting with S19p of the 40S particle, and it is contacting the A-site bound tRNA in yeast (Spahn et al., 2001). However, in comparison to the yeast and human ribosomes, it can be proposed that the modifications in this helix are involved in the structural stabilization of this important subunit-subunit interaction site in Arabidopsis (Karijolich et al., 2010; Sloan et al., 2017).

Further, helix 35 carries one mapped 2′-O-ribose-me site and two pseudouridylation sites at U892 and U899. For the latter two sites guiding snoRNAs were not discovered (Figure 4 and Supplementary Table 2), leading to the assumption that stand-alone enzymes may be responsible. In yeast, helix 35 carries two 2′-O-ribose-me sites and in humans one pseudouridylation site (Piekna-Przybylska et al., 2007). A second intersubunit bridge is formed by helix 34 with the 40S subunit. Thus, also in this case the modifications of helix 35 in plants are likely involved in the stabilization of the neighboring structural element.

In domain III, a high density of modifications is present in the region of helix 47, 50, 59a, and 60 (Figure 4). In yeast, it is assumed that Nop4 is binding to helices 47, 32, 26, 33 but also to helix 60 bringing domain II and III in proximity (Granneman et al., 2011). However, helix 47 in yeast carries no modifications, while Arabidopsis helix 47 is highly modified. It can be speculated that these modifications are required for proper processing of 25S precursors like 27SB or 27S-A_2_/27S-A_3_.

Domains IV (Figure 6) and V (Figure 7) of the 25S rRNA contain the highest degree of modifications. In both domains a total of 66 sites for 2′-O-ribose-me sites are annotated from which 53 could be successfully confirmed. However, for 13 sites no snoRNA could be identified (Figures 6, 7 and Supplementary Table 2). In contrast, 31 pseudouridylation sites were mapped, while seven sites could not be verified yet. For 13 of the mapped sites, the associated snoRNA is not known (Figures 6, 7 and Supplementary Table 2).

The secondary structure map of the core region of 25S rRNA of *A. thaliana* points to a high density of modifications surrounding the PTC (Figure 7), which parallels findings for other organisms (Decatur and Fournier, 2002). The PTC is required for the peptide bond formation and peptide release (Lilley, 2001; Polacek and Mankin, 2005; Torres de Farias et al., 2017). In yeast, defective rRNA modifications in this region lead to increased sensitivity to translational inhibitors or changes in translational fidelity (Baxter-Roshek et al., 2007). In Arabidopsis, especially the helices H73, H74, H75, H88, H89, H90, H91, H92, and H93 contain the highest density of modifications (Figure 7). Interestingly, Arabidopsis contains the highest number of modification sites (34) in these particular helices in contrast to yeast (16) and human (22). This leads to the conclusion that these modifications are of prime importance for the stability of the PTC structure. Nevertheless, Arabidopsis 60S subunit seems to be closer related to the human 60S regarding the high density of modifications. In human ribosomes, helix 74 is important for the accurate structure of the nascent polypeptide exit tunnel (NPET) (Wilson et al., 2020) and helix 93 is a contact site for hydroxylated uL2, which induces structural rearrangements in the PTC of the mature ribosomes (Yanshina et al., 2015). The same could hold true for plants as well. The tip of helix 89 interacts with the GTPase-associated center which might depend on the modifications (Figure 5; Sergiev et al., 2005; Baxter-Roshek et al., 2007) and the modifications in helix 92 were found to be necessary for the correct folding of helix 90–92 in yeast (Baxter-Roshek et al., 2007).

In domain IV, Helix 68, 69, and 71 (Figure 6), are involved in the inter-subunit bridge formation between the 40S and 60S (Spahn et al., 2001; Gigova et al., 2014). Helix 68 contains three mapped methylation and one pseudouridylation sites (Figure 6). One 2′-O-ribose-me site (Am 2210) is conserved in yeast (Am2220) and human (Am3703; Piekna-Przybylska et al., 2007; Supplementary Table 2). The yeast helix 68 contains two E-sites (exit sites), which most probably exist in *A. thaliana* as well (Xie et al., 2012).

Helix 69 is highly modified with two mapped pseudouridylation sites and one mapped 2′-O-ribose-me site in *A. thaliana* (Figure 6). This helix interacts with the tRNAs located in the A and P-site, respectively (Ge and Yu, 2013). Similarly, a cluster of modifications is localized in helix 69 in the yeast rRNA, and their deletions led to e.g., severe growth phenotypes and a lower translational rate (Liang et al., 2007). Helix 71 contains two mapped 2′-O-ribose-me sites at Cm2283 and Gm2278 (Figure 6 and Supplementary Table 2). The site Cm2283 was newly identified but the snoRNA targeting this region was not found (Azevedo-Favory et al., 2020). However, while the human rRNA is lacking this modification, it is conserved between yeast and plants (Piekna-Przybylska et al., 2007).

Interestingly, the enigmatic exceptionally large expansion segment 27 (ES27, Figure 6) was recently unveiled as essential for translational fidelity, in which it seems to regulate amino acid incorporation and by that prevents frameshift errors (Fujii et al., 2018). Furthermore, it was found that this very flexible region of the eukaryotic ribosomes serves as a scaffold for the conserved enzyme methionine amino peptidase (MetAP), which is required to remove co-translationally the first methionine from the nascent polypeptide chain (Fujii et al., 2018; Knorr et al., 2019). Just recently one pseudouridylation site (Ψ2028) without known snoRNA was found in ES27 of Arabidopsis (Sun et al., 2019).

The P-loop in helix 80 and the A-loop in helix 92 are direct pairing sites for A- and P-site tRNA (Kim and Green, 1999). While the P-loop contains one confirmed 2′-O-ribose-methylation site, the A-loop contains two mapped 2′-O-ribose-me sites with the site Gm2912 having a known snoRNA targeting this region. Moreover, the A-loop contains a pseudouridylation site (Figure 7).

Domain VI containing the 3′-end of 25S rRNA from nucleotide 2986 to 3375 contains the conserved sarcin/ricin loop (S/R-Loop; Figure 7). This loop is the site of attack of the two toxins α-sarcin, which is a ribonuclease produced by a fungus and ricin, which is an RNA N-glycosylase synthesized by plants (Endo et al., 1988; Macbeth and Wool, 1999). The attack inhibits proper binding of the elongation factors, and thus, translation is blocked (Szewczak and Moore, 1995). In human 60S subunits the S/L-Loop shows a high degree of modifications in comparison to plants or yeast (Figure 7; Piekna-Przybylska et al., 2007).

Domain VI harbors the lowest degree of modifications with two mapped 2′-O-ribose-me sites with associated snoRNAs and two mapped pseudouridylations sites in Arabidopsis. The two 2′-O-me sites are within helix 100, one pseudouridylation sites in H97 and one in H98 of ES39 (Figure 7). ES39 is exposed to the ribosome surface, the exact function remains elusive, however due to its presence in all eukaryotes it is obvious that eukaryotic ribosomes require this segment (Nygård et al., 2006). For the two pseudouridylation sites U3177 and U3100 a snoRNA is not known so far. Interestingly, the Arabidopsis U3100 is conserved in the human 28S rRNA (U4659), while yeast has not even one modification regarding this specific region (Piekna-Przybylska et al., 2007; Supplementary Table 2).

### A View on the Modifications in Plant 18S rRNA

**FIGURE 8:**
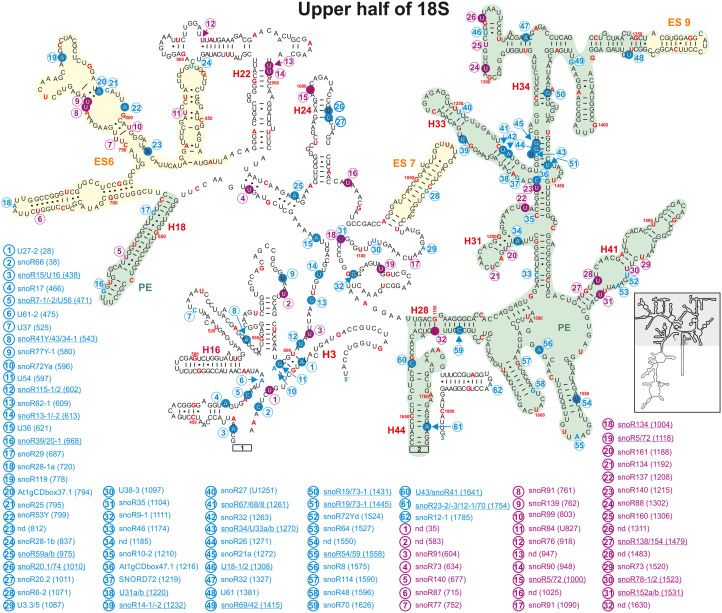
Secondary structure diagram of the 18S rRNA (At2g01010) of *A thaliana* according to The RNAcentral Consortium (2019). Depicted are the positions from 1 to 41, 436 to 1655, and 1750 to 1804. Predicted and verified positions for 2′-O-ribose-methylations and pseudouridylations, as well as of snoRNAs were obtained from Brown et al. (2001, 2003), Qu et al. (2001), Yoshihama et al. (2013), Sun et al. (2019), Streit et al. (2020). The coloring is according to Figure 3. Framed large numbers indicate the position of the connections in Figure 9. PE annotates a pivoting element and ES expansion elements previously identified (Paci and Fox, 2015). The number in brackets correspond to the modified nucleotide position. A small illustration of the whole 40S rRNA secondary structure highlighting the according region is enclosed.

**FIGURE 9:**
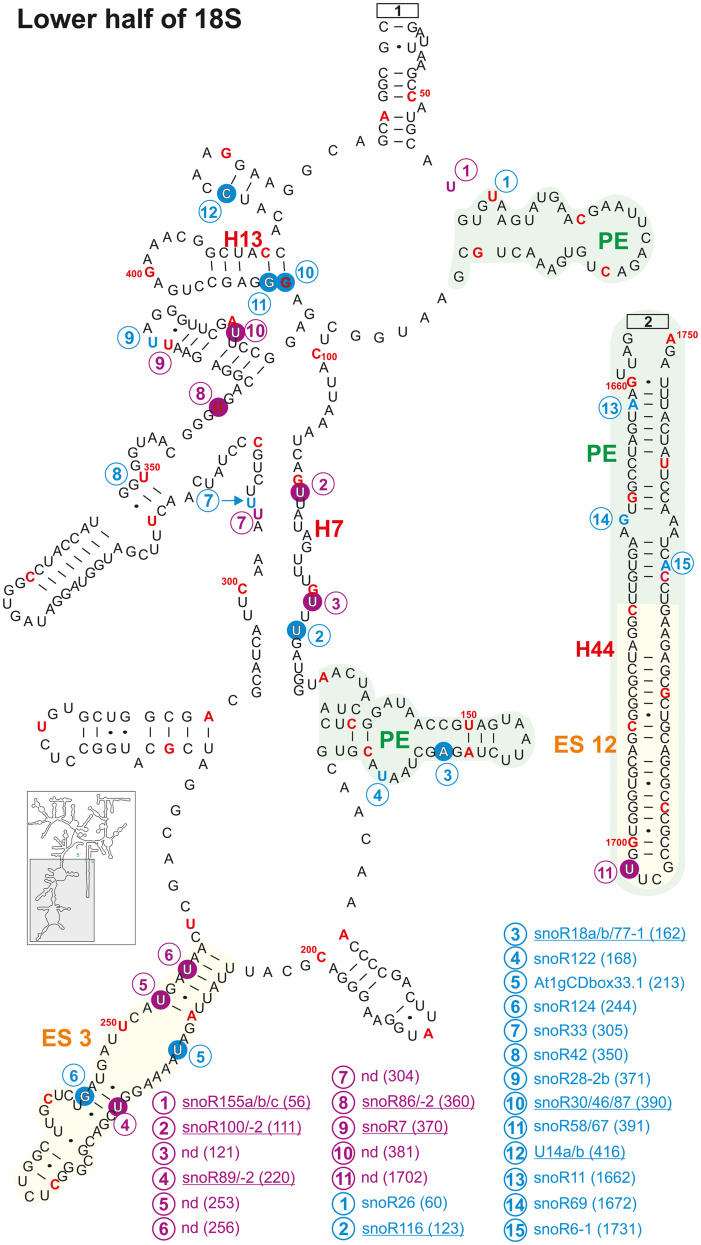
Continuation of the secondary structure diagram of the 18S rRNA (At2g01010) of *A. thaliana* according to The RNAcentral Consortium (2019). All informations are listed in the legend of Figure 8. The number in brackets correspond to the modified nucleotide position. A small illustration of the whole 40S rRNA secondary structure highlighting the according region is enclosed.

The *A. thaliana* genome encodes for two different 18S rRNA variants. While the 18S gene on chromosome 3 has a size of 1808 nt, the copies on chromosomes 2 and 4 contain 1804 nt. The secondary structure model of 18S in here refers to the gene in chromosomes 2 and 4, respectively (Figures 8, 9). The SSU binds the mRNA to decode the genetic information in the “decoding center‘’ (Schluenzen et al., 2000). For the 18S rRNA in total 79 sites are predicted to be 2′-O-ribose methylated, of which 44 are experimentally verified (Supplementary Table 3). Similarly, from 64 predicted pseudouridylation sites 28 were experimentally confirmed.

It is proposed that the decoding center within the SSU consists of the helices 18, 24, 31, 34, and 44 (Liang et al., 2009), which harbor many modifications in yeast. Helix 24 carries the same Ψ-modification in yeast, human and *A. thaliana*. The modifications in helix 18 vary between two 2′-O-ribose-me and one Ψ-site in human, one 2′-O-ribose-me site in yeast and just one Ψ-site in *A. thaliana* (Figure 8), while no methylation site exists in *A. thaliana*.

Helix 34 contains four mapped 2′-O-ribose-me sites in *A. thaliana*, while human and yeast contain three 2′-O-ribose-me sites. Both yeast and human 18S rRNA contain three pseudouridine sites in helix 31 with one site having the hypermodification *m1acp3*Ψ (N1-methyl-N3-aminocarboxypropyl-pseudouridine) at U1191 and U1248, respectively. *A. thaliana* contains two predicted Ψ-sites in this helix, while one of these sites U1192 is the yeast and human equivalent of *m1acp3*Ψ (Piekna-Przybylska et al., 2007). However, it needs to be analyzed whether the plant rRNA also carries hypermodifications as their vertebrate and yeast counterparts.

In helix 44 of the *A. thaliana*, two 2′-O-ribose-me are mapped and additional three are predicted, while only a single Ψ-site is annotated (Figures 8, 9). In contrast, human 18S rRNA contain two and yeast only one 2′-O-ribose-me sites in H44 (Piekna-Przybylska et al., 2007). Interestingly, *A. thaliana* harbors a novel and unique Ψ-site at U1702 in the helix 44 region of ES12 (Figure 9). In yeast, H44 just close to the 3′ end is one of the binding sites for the helicase Prp43, which is required for final maturation of 20S and 27S pre-rRNA, respectively (Bohnsack et al., 2009; Pertschy et al., 2009). For example, in the case of 20S maturation in yeast, Prp43 is assumed to be involved in unwinding of the pre-rRNA enabling endonuclease Nob1 for cleavage at site D (Figure 1; Bohnsack et al., 2009). It is likely, the modification at Am1754 could be necessary for binding of the Prp43 helicase. Yeast, of all things lacks this modification in H44 (Piekna-Przybylska et al., 2007).

Expansion segment 6 (ES6) is located at the surface of the small subunit and highly conserved in plants, vertebrates, and yeast (Alkemar and Nygård, 2003, 2006). As longest expansion segment it contains two mapped Ψ-sites, two mapped 2′-O-ribose-me sites and five predicted sites in *A. thaliana* (Figure 8). In contrast, the rRNA of human ES6 has six Ψ-sites and two 2′-O-ribose-me sites, while the same element in yeast contains two Ψ-sites and one 2′-O-ribose-me site (Piekna-Przybylska et al., 2007). Remarkably, Arabidopsis ES6 shows similarities to both human and yeast. The two Ψ-sites found in *A. thaliana* (Ψ761 and Ψ762) are conserved with the human sites (Ψ814 and Ψ815) and that one of the Arabidopsis 2′-O-ribose-me site (Am799) is conserved to the yeast site (Am796; Figure 8, Piekna-Przybylska et al., 2007; Supplementary Table 3). However, the function of this segment and thus, their modifications remain elusive. *In vivo* crosslinking in yeast suggested that snR30/U17 snoRNAs can bind to two conserved sites in the ES6 to permit proper 18S rRNA processing (Fayet-Lebaron et al., 2009). As snR30 and U17 could not be identified so far in *A. thaliana*, independent pathway for modification might have evolved for *A. thaliana*.

Ribosomal proteins and RBFs bind to specific regions within the rRNA. It could be shown that Enp1 (essential nuclear protein 1) binds to an AUU sequence in helix 33 in yeast, where it is required for pre-rRNA processing of 18S (Chen W. et al., 2003; Granneman et al., 2010). Helix 33 in *Arabidopsis* contains two 2′-O-ribose-methylations (Um1261 and Am1263) in the downstream adjacent region of the AUU site (Figure 8 and Supplementary Table 3). This seems to be unique for plants as yeast and human 18S rRNA lack these modifications (Piekna-Przybylska et al., 2007). Besides, a new modification site at Cm1219 in helix 33 was predicted based on the identification of the snoRNA SNORD72 (Streit et al., 2020).

Helix 41 of the 18S rRNA in *A. thaliana* contains two mapped Ψ-sites at U1483 and U1531 located within a region, which is a binding site for rpS5 in yeast (Figure 8; Supplementary Table 3; Granneman et al., 2010). However, human and yeast helix 41 of the 18S rRNA is not modified (Piekna-Przybylska et al., 2007).

Helix 28 of *A. thaliana* 18S rRNA contains a single mapped 2′-O-ribose-me site at Cm1626 targeted by snoR70 and a single novel Ψ-site at U1630 targeted from an unknown snoRNA (Figure 8). Human 18S rRNA contains a Ψ-site at the same position (U1692) in helix 28, whereas yeast lacks any modification is this region (Piekna-Przybylska et al., 2007; Supplementary Table 3). The helices 36 and 37 of *A. thaliana* contain each one mapped Ψ-site at U1302 (H36, snoR88) and U1311 (H37, unknown snoRNA; Figure 8). Although the human helix 37 contains at least the counterpart of U1311 of Arabidopsis at U1367, yeast helices are absent of these modification sites (Piekna-Przybylska et al., 2007; Supplementary Table 3).

## Conclusion

Prediction and experimental verification suggest that the rRNA of *A. thaliana* is extensively decorated with different varieties of modifications, where pseudouridylations and 2′-O-ribose-methylations represent most of the modifications (Charette and Gray, 2000; Dimitrova et al., 2019). For Arabidopsis 18S rRNA almost 55% of all predicted 2′-O-ribose-methylation sites and 65% of all predicted pseudouridylation sites were successfully experimentally verified (Supplementary Table 3). For the 25S rRNA even 65% of all predicted 2′-O-ribose-methylation sites and almost 58% of predicted pseudouridylation sites were experimentally confirmed (Supplementary Table 2). In contrast, the 5.8S rRNA carries only a low number of modifications (Figure 3 and Supplementary Table 1). Two new modification sites at Ψ22, which is plant specific and Ψ78 (yeast Ψ73) were found in the past (Sun et al., 2019; Supplementary Table 1). In contrast, based on the antisense element of the snoRNA snoR4a/4b (Brown et al., 2001) the modification at the 3′-end of 5.8S rRNA (Gm155) was predicted, but could not be experimentally confirmed (Supplementary Table 1).

The absence of experimental confirmation of predicted sites can have three different reasons. (i) Although nowadays a huge repertoire of techniques is used for mapping of modification sites, a certain limitation in detection sensitivity still exists. An interesting technique would be the use of mung bean nuclease protection assay coupled to RP-HPLC (Yang et al., 2016). (ii) It is discussed those modifications of the rRNA can be tissue or development specific (Chen and Wu, 2009; Sloan et al., 2017; Streit et al., 2020). Thus, the absence of detection can be the result of the analysis of a specific type of ribosomal systems. (iii) It is known that snoRNAs are also involved in the folding of rRNA elements (Bertrand and Fournier, 2013). Hence, it cannot be excluded at stage that some of the modification sites predicted by the detection of snoRNAs might not exist, as the snoRNA is required for guiding a snoRNP involved in rRNA processing or folding.

The modifications in 5.8S varies between the three model species. While yeast 5.8S rRNA contains only one modification site in H7, human and plant 5.8S rRNAs carry two 2′-O-ribose-me and two pseudouridylations (Figure 3; Supplementary Table 1; Piekna-Przybylska et al., 2007). Modifications like pseudouridylations in rRNA are required for the stability of the RNA structure in the ribosomes and 2′-O-ribose-me are necessary for translational accuracy and efficiency (Wu et al., 2015; Erales et al., 2017). Consistent with this finding, the 5.8S rRNA plays a crucial role in translation elongation (translocation; Elela and Nazar, 1997), for which the modifications of 5.8S might be important. In turn, it appears that the 5.8S rRNA of human and plants share high similarities while yeast seems to have evolved a unique way for keeping the structural and translational balance.

Remarkably, human and *A. thaliana* rRNAs share many conserved sites, which are not present in yeast. This elucidates that plant and human rRNA, despite the different sizes, are closer related than plant to yeast rRNA. Moreover, a subset of modifications is clearly unique to Arabidopsis like modifications in the GTPase center (Figure 5), in ES27 (Figure 6) or ES3 in the 18S rRNA (Figure 9). All these regions are targets of a subset of RBFs and RPs. Hence, the plant specific modification pattern stands in relation to the observed plant specificities of the rRNA processing (Weis et al., 2015b; Sáez-Vásquez and Delseny, 2019; Palm et al., 2019) and the modifications might be required for stabilizing the binding of the rRNA to proteins.

Furthermore, alternative functions of snoRNAs were proposed for the plant system. It was suggested that snoRNAs may regulate the modification level of rRNAs and snRNAs under stress as found for drought stress (Zheng et al., 2019). Thus, snoRNAs might have additional functions in plants, which must be discovered.

In future, it will be important to identify the snoRNAs responsible for certain newly discovered modifications sites, and in turn to map rRNA modifications in ribosomes isolated form different tissues, from plants at different developmental stages and after various stress treatments to complete the picture of the Arabidopsis rRNA modification landscape. The latter would perhaps show whether the high number of predicted modifications sites argues for a high ribosome heterogeneity. This concept might even be valid for ribosomes within a single cell but required for the translation of different mRNA pools, which begs the analysis of the rRNA modification profile associated with different mRNAs. Moreover, it will be important to establish a complete profile of rRNA modifications of other plants to allow conclusions on globally conserved and species-specific modifications. The latter is of particular importance to transfer the knowledge based on model systems into agricultural applications.

## Author Contributions

DS and ES: conceptualization, data curation, writing – original draft preparation, and visualization. ES: supervision and funding acquisition. Both authors have read and agreed to the published version of the manuscript.

## Conflict of Interest

The authors declare that the research was conducted in the absence of any commercial or financial relationships that could be construed as a potential conflict of interest.

## Publisher’s Note

All claims expressed in this article are solely those of the authors and do not necessarily represent those of their affiliated organizations, or those of the publisher, the editors and the reviewers. Any product that may be evaluated in this article, or claim that may be made by its manufacturer, is not guaranteed or endorsed by the publisher.

## Supplementary Material

Supplementary Table 1 List of all mapped and predicted modification sites within 5.8S rRNA in *Arabidopsis thaliana* and corresponding human and yeast sites. Not determined (nd).

Supplementary Table 2 List of all mapped or predicted modification sites within 25S rRNA in *Arabidopsis thaliana* and corresponding human and yeast sites. Not determined (nd).

Supplementary Table 3 List of all predicted and mapped modification sites within 18S rRNA in *Arabidopsis thaliana* and corresponding human and yeast sites. Not determined (nd).

## References

[B1] Alkemar G. Nygård O. (2006). Probing the secondary structure of expansion segment ES6 in 18S ribosomal RNA. *Biochemistry* 45 8067–8078. 10.1021/bi052149z 16800631

[B2] Alkemar G. Nygård O. D. D. (2003). A possible tertiary rRNA interaction between expansion segments ES3 and ES6 in eukaryotic 40S ribosomal subunits. *RNA* 9 20–24. 10.1261/rna.2108203 12554872 PMC1370366

[B3] Armache J. P. Jarasch A. Anger A. M. Villa E. Becker T. Bhushan S. (2010a). Cryo-EM structure and rRNA model of a translating eukaryotic 80S ribosome at 5.5-Å resolution. *PNAS* 107 19748–19753. 10.1073/pnas.1009999107 20980660 PMC2993355

[B4] Armache J. P. Jarasch A. Anger A. M. Villa E. Becker T. Bhushan S. (2010b). Localization of eukaryote-specific ribosomal proteins in a 5.5-Å cryo-EM map of the 80S eukaryotic ribosome. *PNAS* 107 19754–19759. 10.1073/pnas.1010005107 20974910 PMC2993421

[B5] Azevedo-Favory J. Gaspin C. Ayadi L. Montacié C. Marchand V. Jobet E. (2020). Mapping rRNA 2′-O-methylations and identification of C/D snoRNAs in Arabidopsis thaliana plants. *RNA Biol.* 1–18. 10.1080/15476286.2020.1869892 33596769 PMC8583080

[B6] Bachellerie J. P. Cavaillé J. (1997). Guiding ribose methylation of rRNA. *Trends Biochem. Sci.* 22 257–261. 10.1016/s0968-0004(97)01057-8 9255067

[B7] Barneche F. Gaspin C. Guyot R. Echeverrìa M. (2001). Identification of 66 box C/D snoRNAs in Arabidopsis thaliana: extensive gene duplications generated multiple isoforms predicting new ribosomal RNA 2′-O-methylation sites. *J. Mol. Biol.* 311 57–73. 10.1006/jmbi.2001.4851 11469857

[B8] Barneche F. Steinmetz F. Echeverrìa M. (2000). Fibrillarin genes encode both a conserved nucleolar protein and a novel small nucleolar RNA involved in ribosomal RNA methylation in arabidopsis thaliana. *J. Biol. Chem.* 275 27212–27220. 10.1016/s0021-9258(19)61499-7 10829025

[B9] Bassham D. C. MacIntosh G. C. (2017). Degradation of cytosolic ribosomes by autophagy-related pathways. *Plant Sci.* 262 169–174. 10.1016/j.plantsci.2017.05.008 28716412

[B10] Baxter-Roshek J. L. Petrov A. N. Dinman J. D. (2007). Optimization of ribosome structure and function by rRNA base modification. *PLoS One* 2:e174. 10.1371/journal.pone.0000174 17245450 PMC1766470

[B11] Beckmann R. Spahn C. M. Eswar N. Helmers J. Penczek P. A. Sali A. (2001). Architecture of the protein-conducting channel associated with the translating 80S ribosome. *Cell* 107 361–372. 10.1016/s0092-8674(01)00541-4 11701126

[B12] Bertrand E. Fournier M. J. (2013). *The snoRNPs and related machines: ancient devices that mediate maturation of rRNA and other RNAs. In Madame Curie Bioscience Database [Internet].* Austin, TX: Landes Bioscience.

[B13] Bhattacharya D. P. Canzler S. Kehr S. Hertel J. Grosse I. Stadler P. F. (2016). Phylogenetic distribution of plant snoRNA families. *BMC Genom.* 17:969. 10.1186/s12864-016-3301-2 27881081 PMC5122169

[B14] Bohnsack M. T. Martin R. Granneman S. Ruprecht M. Schleiff E. Tollervey D. (2009). Prp43 bound at different sites on the pre-rRNA performs distinct functions in ribosome synthesis. *Mol. Cell* 36 583–592. 10.1016/j.molcel.2009.09.039 19941819 PMC2806949

[B15] Briones E. Briones C. Remacha M. Ballesta J. P. (1998). The GTPase Center Protein L12 is required for correct ribosomal stalk assembly but Not for *Saccharomyces* cerevisiae Viability. *J. Biol. Chem.* 273 31956–31961. 10.1074/jbc.273.48.31956 9822666

[B16] Brown J. W. Clark G. P. Leader D. J. Simpson C. G. Lowe T. O. D. D. (2001). Multiple snoRNA gene clusters from Arabidopsis. *RNA* 7 1817–1832. 11780637 PMC1370220

[B17] Brown J. W. Echeverria M. Qu L. H. (2003). Plant snoRNAs: functional evolution and new modes of gene expression. *Trends Plant Sci.* 8 42–49. 10.1016/s1360-1385(02)00007-9 12523999

[B18] Brown J. W. Shaw P. J. (1998). Small nucleolar RNAs and pre-rRNA processing in plants. *Plant Cell* 10 649–657. 10.2307/3870654 9596627 PMC1464647

[B19] Charette M. Gray M. W. (2000). Pseudouridine in RNA: what, where, how, and why. *IUBMB Life* 49 341–351. 10.1080/152165400410182 10902565

[B20] Chen C. L. Liang D. Zhou H. Zhuo M. Chen Y. Q. Qu L. H. (2003). The high diversity of snoRNAs in plants: identification and comparative study of 120 snoRNA genes from Oryza sativa. *Nucleic Acids Res.* 31 2601–2613. 10.1093/nar/gkg373 12736310 PMC156054

[B21] Chen H. M. Wu S. H. (2009). Mining small RNA sequencing data: a new approach to identify small nucleolar RNAs in Arabidopsis. *Nucleic Acids Res.* 37:e69. 10.1093/nar/gkp225 19357091 PMC2685112

[B22] Chen W. Bucaria J. Band D. A. Sutton A. Sternglanz R. (2003). Enp1, a yeast protein associated with U3 and U14 snoRNAs, is required for pre-rRNA processing and 40S subunit synthesis. *Nucleic Acids Res.* 31 690–699. 10.1093/nar/gkg145 12527778 PMC140510

[B23] Chow C. S. Lamichhane T. N. Mahto S. K. (2007). Expanding the nucleotide repertoire of the ribosome with post-transcriptional modifications. *ACS Chem. Biol.* 2 610–619. 10.1021/cb7001494 17894445 PMC2535799

[B24] Decatur W. A. Fournier M. J. (2002). rRNA modifications and ribosome function. *Trends Biochem. Sci.* 27 344–351. 10.1016/s0968-0004(02)02109-6 12114023

[B25] Demirci H. Murphy F. Belardinelli R. Kelley A. C. Ramakrishnan V. Gregory S. T. (2010). Modification of 16S ribosomal RNA by the KsgA methyltransferase restructures the 30S subunit to optimize ribosome function. *RNA* 16 2319–2324. 10.1261/rna.2357210 20962038 PMC2995393

[B26] Desaulniers J. P. Chang Y. C. Aduri R. Abeysirigunawardena S. C. SantaLucia J. Jr. Chow C. S. (2008). Pseudouridines in rRNA helix 69 play a role in loop stacking interactions. *Org. Biomol. Chem.* 6 3892–3895. 10.1039/b812731j 18931791

[B27] Dieci G. Preti M. Montanini B. (2009). Eukaryotic snoRNAs: a paradigm for gene expression flexibility. *Genomics* 94 83–88. 10.1016/j.ygeno.2009.05.002 19446021

[B28] Dimitrova D. G. Teysset L. Carré C. (2019). RNA 2′-O-methylation (Nm) modification in human diseases. *Genes* 10:117. 10.3390/genes10020117 30764532 PMC6409641

[B29] Dragon F. Lemay V. Trahan C. (2006). snoRNAs: biogenesis, structure and function. *Encycl. Life Sci.* 2006 1–7. 10.1002/9780470015902.a0001377

[B30] Egebjerg J. Douthwaite S. R. Liljas A. Garrett R. A. (1990). Characterization of the binding sites of protein L11 and the L10.(L12) 4 pentameric complex in the GTPase domain of 23 S ribosomal RNA from *Escherichia coli*. *J. Mol. Biol.* 213 275–288. 10.1016/s0022-2836(05)80190-1 1692883

[B31] Elela S. A. Nazar R. N. (1997). Role of the 5.8 S rRNA in ribosome translocation. *Nucleic Acids Res.* 25 1788–1794. 10.1093/nar/25.9.1788 9108162 PMC146658

[B32] Endo Y. Chan Y. L. Lin A. Tsurugi K. Wool I. G. (1988). The cytotoxins alpha-sarcin and ricin retain their specificity when tested on a synthetic oligoribonucleotide (35-mer) that mimics a region of 28 S ribosomal ribonucleic acid. *J. Biol. Chem.* 263 7917–7920. 10.1016/s0021-9258(18)68418-2 3372511

[B33] Erales J. Marchand V. Panthu B. Gillot S. Belin S. Ghayad S. E. (2017). Evidence for rRNA 2′-O-methylation plasticity: Control of intrinsic translational capabilities of human ribosomes. *PNAS* 114 12934–12939. 10.1073/pnas.1707674114 29158377 PMC5724255

[B34] Falaleeva M. Welden J. R. Duncan M. J. Stamm S. (2017). C/D-box snoRNAs form methylating and non-methylating ribonucleoprotein complexes: Old dogs show new tricks. *Bioessays* 39:1600264. 10.1002/bies.201600264 28505386 PMC5586538

[B35] Fayet-Lebaron E. Atzorn V. Henry Y. Kiss T. (2009). 18S rRNA processing requires base pairings of snR30 H/ACA snoRNA to eukaryote-specific 18S sequences. *EMBO J.* 28 1260–1270. 10.1038/emboj.2009.79 19322192 PMC2664660

[B36] Ferretti M. B. Karbstein K. (2019). Does functional specialization of ribosomes really exist? *RNA* 25 521–538. 10.1261/rna.069823.118 30733326 PMC6467006

[B37] Filippova J. A. Semenov D. V. Juravlev E. S. Komissarov A. B. Richter V. A. Stepanov G. A. (2017). Modern approaches for identification of modified nucleotides in RNA. *Biochem Mosc.* 82 1217–1233. 10.1134/s0006297917110013 29223150

[B38] Fujii K. Susanto T. T. Saurabh S. Barna M. (2018). Decoding the function of expansion segments in ribosomes. *Mol. Cell* 72 1013–1020. 10.1016/j.molcel.2018.11.023 30576652 PMC6407129

[B39] Ge J. Yu Y. T. (2013). RNA pseudouridylation: new insights into an old modification. *Trends Biochem. Sci.* 38 210–218. 10.1016/j.tibs.2013.01.002 23391857 PMC3608706

[B40] Gigova A. Duggimpudi S. Pollex T. I. M. Schaefer M. Koš M. (2014). A cluster of methylations in the domain IV of 25S rRNA is required for ribosome stability. *RNA* 20 1632–1644. 10.1261/rna.043398.113 25125595 PMC4174444

[B41] Graifer D. Molotkov M. Eremina A. Ven’Yaminova A. Repkova M. Karpova G. (2005). The central part of the 5.8 S rRNA is differently arranged in programmed and free human ribosomes. *Biochem. J.* 387 139–145. 10.1042/bj20041450 15527424 PMC1134941

[B42] Granneman S. Petfalski E. Swiatkowska A. Tollervey D. (2010). Cracking pre-40S ribosomal subunit structure by systematic analyses of RNA–protein cross-linking. *EMBO J.* 29 2026–2036. 10.1038/emboj.2010.86 20453830 PMC2892368

[B43] Granneman S. Petfalski E. Tollervey D. (2011). A cluster of ribosome synthesis factors regulate pre-rRNA folding and 5.8 S rRNA maturation by the Rat1 exonuclease. *EMBO J.* 30 4006–4019. 10.1038/emboj.2011.256 21811236 PMC3209772

[B44] Gulay S. P. Bista S. Varshney A. Kirmizialtin S. Sanbonmatsu K. Y. Dinman J. D. (2017). Tracking fluctuation hotspots on the yeast ribosome through the elongation cycle. *Nucleic Acids Res.* 45 4958–4971. 10.1093/nar/gkx112 28334755 PMC5416885

[B45] Halic M. Becker T. Pool M. R. Spahn C. M. Grassucci R. A. Frank J. (2004). Structure of the signal recognition particle interacting with the elongation-arrested ribosome. *Nature* 427 808–814. 10.1038/nature02342 14985753

[B46] Hang R. Wang Z. Deng X. Liu C. Yan B. Yang C. (2018). Ribosomal RNA biogenesis and its response to chilling stress in Oryza sativa. *Plant physiol.* 177 381–397. 10.1104/pp.17.01714 29555785 PMC5933117

[B47] Hellmann E. (2020). How to make an extraordinary machine: SMALL ORGAN4 regulates ribosome biogenesis in plants. *Plant Physiol.* 184 1627–1629. 10.1104/pp.20.01456 33277331 PMC7723117

[B48] Henras A. K. Soudet J. Gerus M. Lebaron S. Caizergues-Ferrer M. Mougin A. (2008). The post-transcriptional steps of eukaryotic ribosome biogenesis. *Cell. Mol. Life Sci.* 65 2334–2359. 10.1007/s00018-008-8027-0 18408888 PMC11131730

[B49] Higa-Nakamine S. Suzuki T. Uechi T. Chakraborty A. Nakajima Y. Nakamura M. (2012). Loss of ribosomal RNA modification causes developmental defects in zebrafish. *Nucleic Acids Res.* 40 391–398. 10.1093/nar/gkr700 21908402 PMC3245925

[B50] Holley C. L. Li M. W. Scruggs B. S. Matkovich S. J. Ory D. S. Schaffer J. E. (2015). Cytosolic accumulation of small nucleolar RNAs (snoRNAs) is dynamically regulated by NADPH oxidase. *J. Biol. Chem.* 290 11741–11748. 10.1074/jbc.m115.637413 25792744 PMC4416874

[B51] Huang C. Karijolich J. Yu Y. T. (2016). Detection and quantification of RNA 2′-O-methylation and pseudouridylation. *Methods* 103 68–76. 10.1016/j.ymeth.2016.02.003 26853326 PMC4921259

[B52] Ito S. Akamatsu Y. Noma A. Kimura S. Miyauchi K. Ikeuchi Y. (2014). A single acetylation of 18 S rRNA is essential for biogenesis of the small ribosomal subunit in *Saccharomyces* cerevisiae. *J. Biol. Chem.* 289 26201–26212. 10.1074/jbc.m114.593996 25086048 PMC4176211

[B53] Karijolich J. Kantartzis A. Yu Y. T. (2010). RNA modifications: a mechanism that modulates gene expression. *Methods Mol. Biol.* 629 1–19. 10.1007/978-1-60761-657-3_1 20387139 PMC4154353

[B54] Kaul S. Koo H. L. Jenkins J. Rizzo M. Rooney T. Tallon L. J. (2000). Analysis of the genome sequence of the flowering plant Arabidopsis thaliana. *Nature* 408 796–815. 10.1038/35048692 11130711

[B55] Khatter H. Myasnikov A. G. Natchiar S. K. Klaholz B. P. (2015). Structure of the human 80S ribosome. *Nature* 520 640–645. 25901680 10.1038/nature14427

[B56] Kim D. F. Green R. (1999). Base-pairing between 23S rRNA and tRNA in the ribosomal A site. *Mol. Cell.* 4 859–864. 10.1016/s1097-2765(00)80395-0 10619032

[B57] Kim S. H. Spensley M. Choi S. K. Calixto C. P. Pendle A. F. Koroleva O. (2010). Plant U13 orthologues and orphan snoRNAs identified by RNomics of RNA from Arabidopsis nucleoli. *Nucleic Acids Res.* 38 3054–3067. 10.1093/nar/gkp1241 20081206 PMC2875012

[B58] Kiss T. (2001). Small nucleolar RNA-guided post-transcriptional modification of cellular RNAs. *EMBO J.* 20 3617–3622. 10.1093/emboj/20.14.3617 11447102 PMC125535

[B59] Kiss T. Marshallsay C. Filipowicz W. (1991). Alteration of the RNA polymerase specificity of U3 snRNA genes during evolution and in vitro. *Cell* 65 517–526. 10.1016/0092-8674(91)90469-f 1826860

[B60] Kiss-László Z. Henry Y. Kiss T. (1998). Sequence and structural elements of methylation guide snoRNAs essential for site-specific ribose methylation of pre-rRNA. *EMBO J.* 17 797–807. 10.1093/emboj/17.3.797 9451004 PMC1170428

[B61] Klinge S. Woolford J. L. (2019). Ribosome assembly coming into focus. *Nat. Rev. Mol. Cell Biol.* 20 116–131. 10.1038/s41580-018-0078-y 30467428 PMC7725133

[B62] Knorr A. G. Schmidt C. Tesina P. Berninghausen O. Becker T. Beatrix B. (2019). Ribosome–NatA architecture reveals that rRNA expansion segments coordinate N-terminal acetylation. *Nat. Struct. Mol. Biol* 26 35–39. 10.1038/s41594-018-0165-y 30559462

[B63] Kruszka K. Barneche F. Guyot R. Ailhas J. Meneau I. Schiffer S. (2003). Plant dicistronic tRNA–snoRNA genes: a new mode of expression of the small nucleolar RNAs processed by RNase Z. *EMBO J.* 22 621–632. 10.1093/emboj/cdg040 12554662 PMC140725

[B64] Lafontaine D. Vandenhaute J. Tollervey D. (1995). The 18S rRNA dimethylase Dim1pis required for pre-ribosomal RNA process-ing in yeast. *Genes Dev.* 9, 2470–2481. 10.1101/gad.9.20.2470 7590228

[B65] Lafontaine D. L. (2015). Noncoding RNAs in eukaryotic ribosome biogenesis and function. *Nat. Struct. Mol. Biol.* 22 11–19. 10.1038/nsmb.2939 25565028

[B66] Lau R. Y. Kennedy T. D. Lane B. G. (1974). Wheat-embryo ribonucleates. III. Modified nucleotide constituents in each of the 5.8 S, 18S and 26S ribonucleates. *Can. J. Biochem.* 52 1110–1123. 10.1139/o74-155 4447901

[B67] Leader D. J. Clark G. P. Watters J. Beven A. F. Shaw P. J. Brown J. W. (1997). Clusters of multiple different small nucleolar RNA genes in plants are expressed as and processed from polycistronic pre-snoRNAs. *EMBO J.* 16 5742–5751. 10.1093/emboj/16.18.5742 9312032 PMC1170205

[B68] Leader D. J. Sanders J. F. Waugh R. Shaw P. Brown J. W. (1994). Molecular characterisation of plant U14 small nucleolar RNA genes: closely linked genes are transcribed as polycistronic U14 transcripts. *Nucleic Acids Res.* 22 5196–5203. 10.1093/nar/22.24.5196 7816606 PMC332060

[B69] Leidig C. Thoms M. Holdermann I. Bradatsch B. Berninghausen O. Bange G. (2014). 60S ribosome biogenesis requires rotation of the 5S ribonucleoprotein particle. *Nat. Commun.* 5 1–8. 10.1038/ncomms4491 24662372

[B70] Liang X. H. Liu Q. Fournier M. J. (2007). rRNA modifications in an intersubunit bridge of the ribosome strongly affect both ribosome biogenesis and activity. *Mol. Cell.* 28 965–977. 10.1016/j.molcel.2007.10.012 18158895

[B71] Liang X. H. Liu Q. Fournier M. J. (2009). Loss of rRNA modifications in the decoding center of the ribosome impairs translation and strongly delays pre-rRNA processing. *RNA* 15 1716–1728. 10.1261/rna.1724409 19628622 PMC2743053

[B72] Lilley D. M. (2001). The ribosome functions as a ribozyme. *Chembiochem.* 2 31–35. 10.1002/1439-7633(20010105)2:1<31::aid-cbic31>3.0.co;2-p 11828423

[B73] Lindsay M. A. Griffiths-Jones S. Lui L. Lowe T. (2013). Small nucleolar RNAs and RNA-guided post-transcriptional modification. *Essays Biochem.* 54 53–77. 10.1042/bse0540053 23829527

[B74] Liu T. T. Zhu D. Chen W. Deng W. He H. He G. (2013). A global identification and analysis of small nucleolar RNAs and possible intermediate-sized non-coding RNAs in Oryza sativa. *Mol. Plant.* 6 830–846. 10.1093/mp/sss087 22986792 PMC3716300

[B75] Macbeth M. R. Wool I. G. (1999). The phenotype of mutations of G2655 in the sarcin/ricin domain of 23 S ribosomal RNA. *J. Mol. Bio.* 285 965–975. 10.1006/jmbi.1998.2388 9918717

[B76] Marker C. Zemann A. Terhörst T. Kiefmann M. Kastenmayer J. P. Green P. (2002). Experimental RNomics: identification of 140 candidates for small non-messenger RNAs in the plant Arabidopsis thaliana. *Curr. Biol.* 12 2002–2013. 12477388 10.1016/s0960-9822(02)01304-0

[B77] Matera A. G. Terns R. M. Terns M. P. (2007). Non-coding RNAs: lessons from the small nuclear and small nucleolar RNAs. *Nat. Rev. Mol. Cell Biol.* 8 209–220. 10.1038/nrm2124 17318225

[B78] Missbach S. Weis B. L. Martin R. Simm S. Bohnsack M. T. Schleiff E. (2013). 40S ribosome biogenesis co-factors are essential for gametophyte and embryo development. *PloS One* 8:e54084. 10.1371/journal.pone.0054084 23382868 PMC3559688

[B79] Natchiar S. K. Myasnikov A. G. Kratzat H. Hazemann I. Klaholz B. P. (2017). Visualization of chemical modifications in the human 80S ribosome structure. *Nature* 551 472–477. 10.1038/nature24482 29143818

[B80] Nazar R. N. Sitz T. O. Busch H. (1975). Tissue specific differences in the 2′-O-methylation of eukaryotic 5.8 S ribosomal RNA. *FEBS Lett.* 59 83–87. 10.1016/0014-5793(75)80346-2 178541

[B81] Nazar R. N. Sitz T. O. Somers K. D. (1980). Cytoplasmic methylation of mature 5.8 S ribosomal RNA. *J. Mol. Biol.* 142 117–121. 10.1016/0022-2836(80)90209-0 7431406

[B82] Newman D. R. Kuhn J. F. Shanab G. M. Maxwell E. S. (2000). Box C/D snoRNA-associated proteins: two pairs of evolutionarily ancient proteins and possible links to replication and transcription. *RNA* 6 861–879. 10.1017/s1355838200992446 10864044 PMC1369963

[B83] Nygård O. Alkemar G. Larsson S. L. (2006). Analysis of the secondary structure of expansion segment 39 in ribosomes from fungi, plants and mammals. *J. Mol. Biol.* 357 904–916. 10.1016/j.jmb.2006.01.043 16473366

[B84] Paci M. Fox G. E. (2015). Major centers of motion in the large ribosomal RNAs. *Nucleic Acids Res.* 43 4640–4649. 10.1093/nar/gkv289 25870411 PMC4482067

[B85] Palm D. Simm S. Darm K. Weis B. L. Ruprecht M. Schleiff E. (2016). Proteome distribution between nucleoplasm and nucleolus and its relation to ribosome biogenesis in Arabidopsis thaliana. *RNA Biol.* 13 441–454. 10.1080/15476286.2016.1154252 26980300 PMC5038169

[B86] Palm D. Streit D. Shanmugam T. Weis B. L. Ruprecht M. Simm S. (2019). Plant-specific ribosome biogenesis factors in Arabidopsis thaliana with essential function in rRNA processing. *Nucleic Acids Res.* 47 1880–1895. 10.1093/nar/gky1261 30576513 PMC6393314

[B87] Parker M. S. Balasubramaniam A. Sallee F. R. Parker S. L. (2018). The expansion segments of 28S Ribosomal RNA extensively match human messenger RNAs. *Front. Genet.* 9:66. 10.3389/fgene.2018.00066 29563925 PMC5850279

[B88] Pertschy B. Schneider C. Gnädig M. Schäfer T. Tollervey D. Hurt E. (2009). RNA helicase Prp43 and its co-factor Pfa1 promote 20 to 18 S rRNA processing catalyzed by the endonuclease Nob1. *J. Biol. Chem.* 284 35079–35091. 10.1074/jbc.m109.040774 19801658 PMC2787369

[B89] Piekna-Przybylska D. Decatur W. A. Fournier M. J. (2007). The 3D rRNA modification maps database: with interactive tools for ribosome analysis. *Nucleic Acids Res.* 36 D178–D183. 17947322 10.1093/nar/gkm855 PMC2238946

[B90] Polacek N. Mankin A. S. (2005). The ribosomal peptidyl transferase center: structure, function, evolution, inhibition. *Crit. Rev. Biochem. Mol. Biol.* 40 285–311. 10.1080/10409230500326334 16257828

[B91] Polikanov Y. S. Melnikov S. V. Söll D. Steitz T. A. (2015). Structural insights into the role of rRNA modifications in protein synthesis and ribosome assembly. *Nat. Struct. Mol. Biol.* 22 342–344. 10.1038/nsmb.2992 25775268 PMC4401423

[B92] Qu G. Kruszka K. Plewka P. Yang S. Y. Chiou T. J. Jarmolowski A. (2015). Promoter-based identification of novel non-coding RNAs reveals the presence of dicistronic snoRNA-miRNA genes in Arabidopsis thaliana. *BMC Genom.* 16:1009. 10.1186/s12864-015-2221-x 26607788 PMC4660826

[B93] Qu L. H. Meng Q. Zhou H. Chen Y. Q. (2001). Identification of 10 novel snoRNA gene clusters from Arabidopsis thaliana. *Nucleic Acids Res.* 29 1623–1630. 10.1093/nar/29.7.1623 11266566 PMC31268

[B94] Ramesh M. Woolford J. L. (2016). Eukaryote-specific rRNA expansion segments function in ribosome biogenesis. *RNA* 22 1153–1162. 10.1261/rna.056705.116 27317789 PMC4931108

[B95] Ramos L. M. G. Smeekens J. M. Kovacs N. A. Bowman J. C. Wartell R. M. Wu R. (2016). Yeast rRNA expansion segments: folding and function. *J. Mol. Biol.* 428 4048–4059. 10.1016/j.jmb.2016.08.008 27521697

[B96] Reddy R. Henning D. Busch H. (1979). Nucleotide sequence of nucleolar U3B RNA. *J. Biol. Chem.* 254 11097–11105. 10.1016/s0021-9258(19)86635-8 500626

[B97] Reddy R. Sitz T. O. Ro-Choi T. S. Busch H. (1974). Two-dimensional polyacrylamide gel electrophoresis separation of low molecular weight nuclear RNA. *Biochem. Biophys. Res. Commun.* 56 1017–1022. 10.1016/s0006-291x(74)80290-1 4363637

[B98] Rodor J. Jobet E. Bizarro J. Vignols F. Carles C. Suzuki T. (2011). AtNUFIP, an essential protein for plant development, reveals the impact of snoRNA gene organisation on the assembly of snoRNPs and rRNA methylation in Arabidopsis thaliana. *Plant J.* 65 807–819. 10.1111/j.1365-313x.2010.04468.x 21261762

[B99] Ryan P. C. Draper D. E. (1991). Detection of a key tertiary interaction in the highly conserved GTPase center of large subunit ribosomal RNA. *PNAS* 88 6308–6312. 10.1073/pnas.88.14.6308 2068110 PMC52072

[B100] Sáez-Vásquez J. Delseny M. (2019). Ribosome biogenesis in plants: from functional 45S ribosomal DNA organization to ribosome assembly factors. *Plant Cell.* 31 1945–1967. 10.1105/tpc.18.00874 31239391 PMC6751116

[B101] Samarsky D. A. Fournier M. J. Singer R. H. Bertrand E. (1998). The snoRNA box C/D motif directs nucleolar targeting and also couples snoRNA synthesis and localization. *EMBO J.* 17 3747–3757. 10.1093/emboj/17.13.3747 9649444 PMC1170710

[B102] Schluenzen F. Tocilj A. Zarivach R. Harms J. Gluehmann M. Janell D. (2000). Structure of functionally activated small ribosomal subunit at 3.3 Å resolution. *Cell* 102 615–623. 10.1016/s0092-8674(00)00084-2 11007480

[B103] Sergiev P. V. Lesnyak D. V. Burakovsky D. E. Kiparisov S. V. Leonov A. A. Bogdanov A. A. (2005). Alteration in location of a conserved GTPase-associated center of the ribosome induced by mutagenesis influences the structure of peptidyltransferase center and activity of elongation factor G. *J. Biol. Chem.* 280 31882–31889. 10.1074/jbc.m505670200 16014631

[B104] Sharma S. Marchand V. Motorin Y. Lafontaine D. L. (2017a). Identification of sites of 2′-O-methylation vulnerability in human ribosomal RNAs by systematic mapping. *Sci. Rep.* 7 1–15. 28904332 10.1038/s41598-017-09734-9 PMC5597630

[B105] Sharma S. Yang J. van Nues R. Watzinger P. Kötter P. Lafontaine D. L. (2017b). Specialized box C/D snoRNPs act as antisense guides to target RNA base acetylation. *PLoS Genet.* 13:e1006804. 10.1371/journal.pgen.1006804 28542199 PMC5464676

[B106] Sloan K. E. Warda A. S. Sharma S. Entian K. D. Lafontaine D. L. Bohnsack M. T. (2017). Tuning the ribosome: The influence of rRNA modification on eukaryotic ribosome biogenesis and function. *RNA Biol.* 14 1138–1152. 10.1080/15476286.2016.1259781 27911188 PMC5699541

[B107] Spahn C. M. Beckmann R. Eswar N. Penczek P. A. Sali A. Blobel G. (2001). Structure of the 80S ribosome from Saccharomyces cerevisiae—tRNA-ribosome and subunit-subunit interactions. *Cell* 107 373–386. 10.1016/s0092-8674(01)00539-6 11701127

[B108] Streit D. Shanmugam T. Garbelyanski A. Simm S. Schleiff E. (2020). The existence and localization of nuclear snoRNAs in Arabidopsis thaliana revisited. *Plants* 9:1016. 10.3390/plants9081016 32806552 PMC7464842

[B109] Sun L. Xu Y. Bai S. Bai X. Zhu H. Dong H. (2019). Transcriptome-wide analysis of pseudouridylation of mRNA and non-coding RNAs in Arabidopsis. *J. Exp. Bot.* 70 5089–5600. 10.1093/jxb/erz273 31173101 PMC6793436

[B110] Szewczak A. A. Moore P. B. (1995). The sarcin/ricin loop, a modular RNA. *J. Mol. Biol.* 247 81–98. 10.1006/jmbi.1994.0124 7897662

[B111] Taylor D. J. Devkota B. Huang A. D. Topf M. Narayanan E. Sali A. (2009). Comprehensive molecular structure of the eukaryotic ribosome. *Structure* 17 1591–1604. 10.1016/j.str.2009.09.015 20004163 PMC2814252

[B112] Terns M. P. Terns R. M. (2002). Small nucleolar RNAs: versatile trans-acting molecules of ancient evolutionary origin. *Gene Expr.* 10 17–39. 11868985 PMC5977530

[B113] The RNAcentral Consortium (2019). RNAcentral: a hub of information for non-coding RNA sequences. *Nucleic Acids Res.* 47 D221–D229. 30395267 10.1093/nar/gky1034 PMC6324050

[B114] Tollervey D. Kiss T. (1997). Function and synthesis of small nucleolar RNAs. *Curr. Opin. Cell Biol.* 9 337–342. 10.1016/s0955-0674(97)80005-1 9159079

[B115] Torres de Farias S. Gaudêncio Rêgo T. José M. V. (2017). Peptidyl transferase center and the emergence of the translation system. *Life* 7:21. 10.3390/life7020021 28441334 PMC5492143

[B116] Tsang C. K. Bertram P. G. Ai W. Drenan R. Zheng X. S. (2003). Chromatin-mediated regulation of nucleolar structure and RNA Pol I localization by TOR. *EMBO J.* 22 6045–6056. 10.1093/emboj/cdg578 14609951 PMC275436

[B117] Turkina M. V. Årstrand H. K. Vener A. V. (2011). Differential phosphorylation of ribosomal proteins in Arabidopsis thaliana plants during day and night. *PloS One* 6:e29307. 10.1371/journal.pone.0029307 22195043 PMC3241707

[B118] Venema J. Tollervey D. (1999). Ribosome synthesis in *Saccharomyces* cerevisiae. *Annu. Rev. Genet.* 33 261–311. 10690410 10.1146/annurev.genet.33.1.261

[B119] Watkins N. J. Bohnsack M. T. (2012). The box C/D and H/ACA snoRNPs: key players in the modification, processing and the dynamic folding of ribosomal RNA. *Wiley Interdiscip. Rev. RNA.* 3 397–414. 10.1002/wrna.117 22065625

[B120] Weinstein L. B. Steitz J. A. (1999). Guided tours: from precursor snoRNA to functional snoRNP. *Curr. Opin. Cell Biol.* 11, 378–384. 10.1016/S0955-0674(99)80053-2 10395551

[B121] Weis B. L. Kovacevic J. Missbach S. Schleiff E. (2015b). Plant-specific features of ribosome biogenesis. *Trends Plant Sci.* 20 729–740. 10.1016/j.tplants.2015.07.003 26459664

[B122] Weis B. L. Palm D. Missbach S. Bohnsack M. T. Schleiff E. (2015a). atBRX1-1 and atBRX1-2 are involved in an alternative rRNA processing pathway in *Arabidopsis thaliana*. *RNA* 21 415–425. 10.1261/rna.047563.114 25605960 PMC4338337

[B123] Wilson D. M. Li Y. LaPeruta A. Gamalinda M. Gao N. Woolford J. L. (2020). Structural insights into assembly of the ribosomal nascent polypeptide exit tunnel. *Nat. commun.* 11 1–15. 33037216 10.1038/s41467-020-18878-8 PMC7547690

[B124] Woolford J. L. Jr. Baserga S. J. (2013). Ribosome biogenesis in the yeast *Saccharomyces* cerevisiae. *Genetics* 195 643–681. 10.1534/genetics.113.153197 24190922 PMC3813855

[B125] Wu G. Huang C. Yu Y. T. (2015). Pseudouridine in mRNA: incorporation, detection, and recoding. *Meth. Enzymol.* 560 187–217. 10.1016/bs.mie.2015.03.009 PMC5702494 26253972

[B126] Xie Q. Wang Y. Lin J. Qin Y. Wang Y. Bu W. (2012). Potential key bases of ribosomal RNA to kingdom-specific spectra of antibiotic susceptibility and the possible archaeal origin of eukaryotes. *PloS One* 7:e29468. 10.1371/journal.pone.0029468 22247777 PMC3256160

[B127] Yang J. Sharma S. Watzinger P. Hartmann J. D. Kötter P. Entian K. D. (2016). Mapping of complete set of ribose and base modifications of yeast rRNA by RP-HPLC and mung bean nuclease assay. *PloS One* 11:e0168873. 10.1371/journal.pone.0168873 28033325 PMC5199042

[B128] Yanshina D. D. Bulygin K. N. Malygin A. A. Karpova G. G. (2015). Hydroxylated histidine of human ribosomal protein uL2 is involved in maintaining the local structure of 28S rRNA in the ribosomal peptidyl transferase center. *FEBS J.* 282 1554–1566. 10.1111/febs.13241 25702831

[B129] Yoshihama M. Nakao A. Kenmochi N. (2013). snOPY: a small nucleolar RNA orthological gene database. *BMC Res. Notes.* 6:426. 10.1186/1756-0500-6-426 24148649 PMC4015994

[B130] Zhao X. Yu Y. T. (2004). Detection and quantitation of RNA base modifications. *RNA* 10 996–1002. 10.1261/rna.7110804 15146083 PMC1370591

[B131] Zhao Y. Yu Y. Zhai J. Ramachandran V. Dinh T. T. Meyers B. C. (2012). The Arabidopsis nucleotidyl transferase HESO1 uridylates unmethylated small RNAs to trigger their degradation. *Curr. Biol.* 22 689–694. 10.1016/j.cub.2012.02.051 22464194 PMC3350747

[B132] Zheng J. Zeng E. Du Y. He C. Hu Y. Jiao Z. (2019). Temporal small RNA expression profiling under drought reveals a potential regulatory role of small nucleolar RNAs in the drought responses of Maize. *Plant Genome* 12 1–15. 10.3835/plantgenome2018.08.0058 PMC12962343 30951096

[B133] Zhou H. Meng Q. Qu L. (2000). Identification and structural analysis of a novel snoRNA gene cluster from Arabidopsis thaliana. *Sci. China C. Life Sci.* 43 449–453.

[B134] Zhu P. Wang Y. Qin N. Wang F. Wang J. Deng X. W. (2016). Arabidopsis small nucleolar RNA monitors the efficient pre-rRNA processing during ribosome biogenesis. *Proc. Natl. Acad. Sci.* 113 11967–11972. 10.1073/pnas.1614852113 27708161 PMC5081647

